# Identification of Genomic Regions Controlling Leaf Scald Resistance in Sugarcane Using a Bi-parental Mapping Population and Selective Genotyping by Sequencing

**DOI:** 10.3389/fpls.2018.00877

**Published:** 2018-06-26

**Authors:** Andres F. Gutierrez, Jeffrey W. Hoy, Collins A. Kimbeng, Niranjan Baisakh

**Affiliations:** ^1^School of Plant, Environmental and Soil Sciences, Louisiana State University Agricultural Center, Baton Rouge, LA, United States; ^2^Department of Plant Pathology and Crop Physiology, Louisiana State University Agricultural Center, Baton Rouge, LA, United States; ^3^Sugar Research Station, Louisiana State University Agricultural Center, St. Gabriel, LA, United States

**Keywords:** genotyping by sequencing, linkage map, leaf scald, marker, QTL, sugarcane

## Abstract

Leaf scald, caused by *Xanthomonas albilineans*, is a major sugarcane disease worldwide. The disease is managed primarily with resistant cultivars obtained through classical breeding. However, erratic symptom expression hinders the reliability and reproducibility of selection for resistance. The development and use of molecular markers associated with incompatible/compatible reactions could overcome this limitation. The aim of the present work was to find leaf scald resistance-associated molecular markers in sugarcane to facilitate marker-assisted breeding. A genetic linkage map was constructed by selective genotyping of 89 pseudo F_2_ progenies of a cross between LCP 85-384 (resistant) and L 99-226 (susceptible) using 1,948 single dose (SD) markers generated from SSR, eSSR, and SNPs. Of these, 1,437 SD markers were mapped onto 294 linkage groups, which covered 19,464 cM with 120 and 138 LGs assigned to the resistant and susceptible parent, respectively. Composite interval mapping identified 8 QTLs associated with the disease response with LOD scores ranging from 3.0 to 7.6 and explained 5.23 to 16.93% of the phenotypic variance. Comparative genomics analysis with *Sorghum bicolor* allowed us to pinpoint three SNP markers that explained 16% phenotypic variance. In addition, representative stress-responsive genes close to the major effect QTLs showed upregulation in their expression in response to the bacterial infection in leaf/meristem tissue.

## Introduction

Sugarcane (*Saccharum* spp. hybrids) is a tropical C4 member of the Poaceae family, which accounts for 70% of the raw sugar produced worldwide (Le Cunff et al., 2008; Aitken et al., 2014). Cultivated sugarcane is derived from inter-specific hybridizations between two polyploid species *Saccharum officinarum* (2*n* = 8× = 80) and *S. spontaneum* (2*n* = 10× = 40–120) (Aitken et al., 2014). The hybridization involved the combination of vigorous growth, tolerance to abiotic stresses and disease resistance from *S. spontaneum* with agronomic characteristics, including high sucrose content from *S. officinarum*. In the development of modern cultivars, the initial hybrids were backcrossed with *S. officinarum* to recover high sucrose content. Thus, the modern cultivars are complex aneu-polyploids with chromosome numbers of 2*n* = 100–120 (D’Hont et al., 1998; Aitken et al., 2014) that constitute approximately 80% of *S. officinarum*, 10–15% of *S. spontaneum*, and 5–10% recombinant chromosomes (D’Hont et al., 1996). The high ploidy level, the aneuploidy and the cytogenetic complexity have made sugarcane a challenge for breeding, genetics, and gene cloning (D’Hont and Glaszmann, 2001; Rossi et al., 2003).

Diseases are one of the most important problems that affect sugarcane productivity (Rott and Davis, 2000). Leaf scald, caused by the bacterium *Xanthomonas albilineans* (Ashby) Dowson, is one of the major diseases worldwide (Wang et al., 1999; Rott and Davis, 2000). The disease is characterized by possible latent, chronic and acute phases varying in severity from a white, sharply defined longitudinal leaf stripe to death of shoots or entire plants (Ricaud and Ryan, 1989; Rott et al., 1997). Leaf scald causes high losses in tons of cane per hectare and reduction in juice quality (Ricaud and Ryan, 1989; Rott and Davis, 2000). Hot water treatment has been shown to partially control leaf scald because of the pathogen’s vascular association. Moreover, management by hot water treatment is considered another significant cost to the industry (Rott and Davis, 2000). Host plant resistance, tissue culture to produce healthy seed-cane, disinfection of cutting and harvesting tools with bactericides, and quarantine measures during germplasm exchanges are methods used to prevent and control the disease (Ricaud and Ryan, 1989; Rott and Davis, 2000).

The development of resistant varieties is considered the best strategy to manage leaf scald in sugarcane. The troublesome aspect of resistance evaluation is that symptom expression is strongly affected by environmental conditions with severe symptom development being associated with the occurrence of drought conditions (Rott et al., 1997; Rott and Davis, 2000). The erratic symptom expression results in the failure to accurately detect susceptibility and thus multiple field trials utilizing inoculation are needed. However, inoculation can result in systemic infection of resistant clones (Gutierrez et al., 2016). Under this scenario, the marker-assisted selection (MAS) technique, which uses DNA marker(s) linked to useful trait(s), would be very useful in breeding for resistance against the disease (Costet et al., 2012a).

The large (10 Gb) and complex genome, the absence of a reference genome draft, the coexistence of single and multi-dose alleles, and the irregular number of chromosomes in the homo(eo)logy groups have hindered progress in the development and application of genetic/genomic tools in sugarcane (Wang et al., 2010). Until recently, all sugarcane genetic maps constructed were incomplete due to the large number of chromosomes and the limited number of markers used for mapping. Moreover, the makers that were used in the past for developing genetic maps are SSRs, EST-derived AFLPs, and DArTs that did not generate enough markers to cover the large sugarcane genome. However, with the decrease in the cost of DNA sequencing technologies, next generation sequencing (NGS)-based genotyping has recently been used to develop high-density molecular maps that are being used in QTL mapping, gene tagging, and map-based cloning (Yang et al., 2017).

The genetic maps developed for sugarcane cultivars, as well as for their ancestral species, are based on populations of full sib (F_1_) individuals following a pseudo-test cross strategy using only single dose markers (Wu et al., 1992; Grattapaglia and Sederoff, 1994). In a bi-parental population, a single dose marker has either a single copy of an allele in one parent segregating in 1:1 (presence:absence) or a single copy of the same allele in both parents segregating in 3:1 (presence:absence). Based on this method, partial genetic maps have been produced for *S. spontaneum* (Da Silva et al., 1993; Ming et al., 1998), *S. officinarum* (Guimaraes et al., 1999; Aitken et al., 2006), interspecific hybrids (Daugrois et al., 1996), and modern cultivars of sugarcane (Hoarau et al., 2001; Andru et al., 2011; Singh et al., 2013; Aitken et al., 2014). However, with the development of NGS and software tools capable of producing and processing millions of sequence variations, restriction enzyme-based genotyping by sequencing method (Elshire et al., 2011) has been used to identify single nucleotide polymorphism (SNP) markers that were used for development of high-density linkage maps in sugarcane (Balsalobre et al., 2017; Yang et al., 2017).

A handful of QTL studies have been conducted in sugarcane reporting the genomic regions that control agronomic traits of interest, including sugar traits (cf. Balsalobre et al., 2017). DNA markers associated with disease resistance were reported for brown rust (Daugrois et al., 1996; Asnaghi et al., 2004; Le Cunff et al., 2008; Costet et al., 2012a; Yang et al., 2017), yellow spot (Aljanabi et al., 2007), yellow leaf virus (Costet et al., 2012b; Debibakas et al., 2014), and downy mildew (Baer and Lalusin, 2013). However, the only QTL that has been fine resolved using synteny-based comparative mapping with sorghum is *Bru*1 for brown rust resistance (Costet et al., 2012a). This led to the development of PCR-based markers linked to *Bru*1 that have been used in MAS in several breeding programs worldwide (Glynn et al., 2013; Racedo et al., 2013; Parco et al., 2014, 2017). The success with *Bru*1 provides an example that MAS is feasible in sugarcane. The present study reports on the identification of QTLs associated with resistance to leaf scald using selective genotyping of a subset of an F_1_ progeny from a bi-parental population developed from the cross between two parents with contrasting disease response.

## Materials and Methods

### Plant Materials

High heterozygosity of sugarcane cultivars makes it possible to use an F_1_ population as a pseudo F_2_ mapping population. The F_1_ progeny derived from the cross between a leaf scald resistant cultivar LCP 85-384 (female) and a susceptible cultivar L 99-226 (male) was used to develop a linkage map. LCP 85-384 and L 99-226 were selected from the progeny of a cross between CP 77-310 and CP 77-407 (Milligan et al., 1994) and HoCP 89-846 and LCP 81-30 (Bischoff et al., 2009), respectively. The seedling progeny of the mapping population was germinated in the greenhouse and transplanted to seedling trays after 3 weeks, and the survivor clones of this process were planted in the field at the Sugar Research Station, St. Gabriel, LA, United States. One hundred and eighty-six individuals randomly selected from the population were used in the study. The population along with the parents was maintained as clones in field plots where each clone represented a single plot 2.4 m long in a completely randomized layout.

### Leaf Scald Reaction Evaluation and Data Analysis

The population (186 F_1_ and parents) was evaluated as plant canes (first year crop) in two growing seasons (2014 and 2015). *Xanthomonas albilineans* isolation and quantification, and plant inoculation by decapitation were performed following the protocols previously described (Garces et al., 2014). Bacterial suspension at a concentration of 3.5 × 10^8^ CFU/μL (0.18 OD at 590 nm) was kept at 4°C in the dark prior to inoculation. Plants (20 biological replicates per clone) were inoculated at sunset by spraying the bacterial suspension on the surface of the shoot cut above the apical meristem with scissors dipped in the inoculum suspension (Koike, 1965). In the summer of 2014, inoculation was performed on June 12. Two inoculations were performed in 2015, in different sugarcane plantings – the first inoculation was performed on May 29 and the second on June 9.

Disease severity was evaluated based on the type of symptoms observed 8 weeks after inoculation in intact leaves that emerged after the inoculation in 6 to 14 stalks per clone. Visual symptom severity was assessed for systemically infected leaves and rated using a 1 to 9 scale where 1–3 was considered to be resistant, 4–6 as moderately susceptible, and 7–9 as highly susceptible. Disease severity was evaluated for each clone using the formula: Resistance rating = [(1 × NS) + (3 × PL) + (5 × ML) + (7 × N) + (9 × D)]/T, where NS = number of stalks without symptoms; PL = number of stalks with leaves exhibiting one or two narrow, white, pencil-line streaks; ML = number of stalks with more than two pencil-line streaks in leaves; N = number of stalks with leaf necrosis or bleaching; D = number of dead stalks or stalks with side shooting; and T = total number of stalks evaluated per clone.

The visual ratings were transformed using the Box-Cox transformation with λ values of -1.2 (2014 data), -0.2 (first set of 2015), and 0.1 (second set 2015) using the formula (y^λ^–1)/λ (if λ ≠ 0). The Box-Cox coefficients (λ) were obtained using SAS software v. 9.3 (SAS Institute Inc., Cary, NC, United States). The transformed data were evaluated for normality using the Shapiro and Wilk test, and heritability was estimated using VARCOMP procedure in SAS software v. 9.3.

### DNA Extraction and Genotyping

Genomic DNA was isolated from freshly collected leaves of the progeny and parents using the potassium acetate protocol (Dellaporta et al., 1983). The DNA samples of parents, grandparents and 89 F_1_ selected based on the disease symptom severity ratings assigned in 2014 (36 resistant, 28 moderate resistant, 16 moderate susceptible, and 9 highly susceptible clones; the samples in each disease reaction group were represented in similar proportions in the original population of 186 progeny) were used for genotyping. DNA quantity and quality were estimated using the Nanodrop 1000 spectrophotometer (NanoDrop, Bethesda, MD, United States).

Genotyping was performed using simple sequence repeat (SSR) as well as SNP markers. A total of 121 SSR primers (mapped on 10 *Sorghum bicolor* chromosomes) from the Sugarcane Microsatellite Consortium (Cordeiro et al., 2000; Pan, 2006) and 31 eSSRs developed from the leaf scald suppressive subtractive hybridization cDNA library (**Supplementary Table S1**) were used. For SSR genotyping, 50 ng of genomic DNA was used as the template in PCR reactions in a final volume of 10 μl containing 1× PCR buffer, 2.5 mM MgCl_2_, 0.2 μM dNTP mix, 0.4 unit of *Taq* DNA polymerase (Promega, Madison, WI, United States), and 0.75 μM of each primer. PCR amplification reactions were conducted on a C1000 Touch Thermal Cycler equipped with a 384 well block (Bio-Rad, Hercules, CA, United States) with a thermal profile of initial denaturation at 95°C for 5 min, 35 cycles at 95°C for 15 s, 58°C for 15 s, and 72°C for 1 min, and a final extension at 72°C for 10 min. PCR products were resolved in 13% polyacrylamide gels using a HEGS electrophoresis apparatus (Nihon Eido, Tokyo, Japan). The gels were stained using ethidium bromide and visualized and documented in a Kodak Gel Logic200 gel documentation system (Carestream, Rochester, NY, United States). The SSRs and eSSRs amplified fragments were manually scored as ‘1’ for presence and ‘0’ for absence.

For genotyping by sequencing, 500 ng of DNA of each sample was used for library preparation as per Elshire et al. (2011). Briefly, DNA was restricted by *Pst*I enzyme and ligated with adapters for barcoding. Barcoded DNA from parents, grandparents, and the progeny were pooled and 96-plex sequenced in a single flow cell on an Illumina HiSeq2500 platform at the Institute of Biotechnology of Cornell University, BRC Genomics Facility, Ithaca, NY, United States.

Clean, filtered sequence reads after removing the adapter and restriction enzyme reminiscent with Phred quality score ≥ 20 were used for SNP calling. Two reference-based SNP callers, GBS Tassel (Glaubitz et al., 2014), and Samtools (Li et al., 2009) were used. In the absence of the sugarcane reference genome, the *Sorghum bicolor* genome (v.3.0), because of its microsynteny with sugarcane (Wang et al., 2010), was used as the reference, and uniquely mapped reads were used for variant calling. SNPs were called from GBS tags that constituted of at least three reads with identical sequence. Samtools pipeline was used as per the default parameters. Only SNPs that were commonly called by both software tools were subjected to a second level of filtering to remove SNPs that were not present in both parents and had more than 10% missing data.

### Marker Segregation Analysis

Mono- and polymorphic fragments were produced by all the marker systems. In sugarcane, several segregation ratios are possible in the F_1_ population. With the assumptions of polysomic inheritance and absence of segregation distortion, single dose (SD) markers are present only once in the genome and they are expected to segregate in 1:1 (present in one parental genome) and 3:1 (present in both parents) (Da Silva et al., 1993). Each marker was tested against expected segregation ratio using a χ^2^ goodness fit test (*df* = 1) at 5% error level (type I) for SD or bi-parental SD segregation ratios.

### Linkage Map Construction

Mapping of the SD markers onto linkage groups was done using OneMap v. 2.0-4 package of R v.3.1.3 (Margarido et al., 2007). The SSR and eSSR markers were mapped as a dominant marker (presence versus absence). The linkage map construction was performed in two steps following the method suggested for polyploid species (Wu et al., 1992). Markers were grouped as D1 (D1.10 and D1.13) originating from LCP 85-384 and D2 (D2.15 and D2.18) from L 99-226, and C8 and B3.7 originating from both parents as described by Wu et al. (2002). Only SD markers were used to build the framework map of each parent with LOD (Log_10_ of odds) score threshold of 4.0 and a recombination fraction value of 0.40. Linkage groups containing only the 3:1 SD markers (C8 and B3.7) belonged to both parental maps. OneMap allows construction of linkage groups carrying markers from both parents (D1 and D2) using 3:1 markers as hinge. Genetic distances between markers were computed using the Kosambi mapping function. To construct the homology group (HG), the markers in LGs were aligned into the sorghum chromosomes. LGs with more than 80% of their markers mapped to a single sorghum chromosome were grouped into one HG. Recombinant linkage groups were formed with markers that were located on different HGs. Linkage groups with significant QTLs with high LOD scores and percentage of phenotypic variance explained (PVE) were selected for saturation. In the saturation process, the SD markers that could not be mapped previously but flanking the QTL regions (based on the genome information of *Sorghum bicolor*) were selected with a less stringent selection (Bonferroni correction was applied in the χ^2^ test) for integration into the map. The graphic representation of the linkage groups was performed using MapChart v.2.3 (Voorrips, 2002).

### QTL Mapping

QTL analysis was performed on the transformed phenotypic data from the three field trials over two crop years, using the Windows QTL Cartographer Software v.2.5 (Wang et al., 2012) and QTL IciMapping Software v.4.1 (Wang et al., 2016). To confirm the location of the QTLs, composite interval mapping (CIM) was undertaken with markers as co-factors selected by forward and backward step-wise regression with 10 cM window size and 1 cM walking speed settings in Win QTL Cartographer v.2.5 (Wang et al., 2012) with 1,000 iterations. A LOD of 2.5 and a 5% PVE were used as the threshold to declare a QTL significant (Churchill and Doerge, 1994).

Based on the microsynteny between sugarcane and sorghum genomes, the location of the markers from QTL analysis were ascertained in the sorghum genome that facilitated the search for the genes flanking/within the QTL regions. Genes located within 20-kb surrounding the QTL regions were considered as candidate genes associated with the resistance response to leaf scald. For validation of the effect of a marker closest to a QTL, allele-specific primers were designed and PCR was run on all 186 F1 progeny as described earlier (Drenkard et al., 2000; Solis et al., 2017). Expression profile of three genes selected in the QTL regions was analyzed using real-time PCR as described earlier (Khan et al., 2013).

## Results

### Leaf Scald Response of the F_1_ Progeny in the Field

**FIGURE 1:**
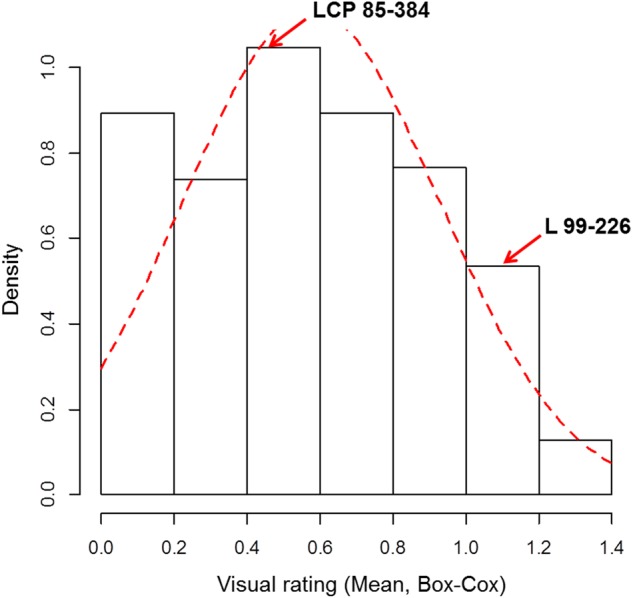
Frequency distribution of the Box-Cox transformed visual rating of leaf scald response under field conditions of F_1_ progeny of LCP 85-384 × L 99-226. Density = frequency/interval.

Leaf scald reaction of the F_1_ population was evaluated visually 8 weeks after inoculation for plant cane in three different trials (one in 2014 and two in 2015) on a scale of 1–9. The phenotypic distribution was not normal and skewed to the left due to the high number of resistant progeny in the F_1_ population. The use of the Box-Cox transformation showed low to intermediate correlation among the three field trials (**Table 1**). In contrast, the correlation among the different trials evaluated with the average of the visual symptom rating was high. Moreover, the transformed data presented a near-normal distribution (**Figure 1**) by Shapiro–Wilk test with *p*-value = 0.4157, *W* = 0.9943, and eliminated the left skewness with the skewness value near to zero (0.086). The heritability in broad sense of the leaf scald reaction (H^2^), based on the severity of symptom expression, was 0.24 per plot and 0.48 per mean (**Supplementary Data Sheet S1**) that showed a low to medium genetic variance component and a high effect of the environment on leaf scald symptom expression.

**Table 1:** Pearson correlation among three field evaluations of leaf scald resistance reaction on the bi-parental F_1_ population of LCP 85-384 × L 99-226.

	Visual rating		
Trials	2014	2015a	2015b
2014	1	0.3486 (*p* = 0.0009)	0.2558 (*p* = 0.0162)
2015a		1	0.3865 (*p* = 0.0002)
2015b			1

*a, 2015 first season; b, 2015, second season.*

The low to medium correlation among the data sets of the three time-point disease reaction evaluation led to the use of all the data sets in the QTL analysis. The QTLs reported in this study were found with at least two of the three field evaluations. The high (visual symptom rating) correlations of the average data with the trials allowed using the average information for the initial QTL mapping.

### Genotyping and Marker Data

A total of 332 unambiguous alleles were obtained with genotyping of the F_1_ progeny using 121 polymorphic SSR markers. Genotyping using 31 polymorphic eSSR markers resulted in 24 scorable alleles. Of these, 202 SSR (60.8%) and 20 eSSR (83.3%) alleles segregated as SD markers by χ^2^ test that were included for linkage mapping. A total of 250,451,013 single-end 100 bp reads were obtained from the GBS of the mapping population and parents of which 225,489,934 were good-barcoded reads. Filtering for barcodes and restriction enzyme remnants produced 209,848,011 reads.

From the genotyping by sequencing of 95 individuals (89 F_1_ individuals plus parents and grandparents), a total of 28,722 and 27,260 SNP markers were called using Samtools and Tassel, respectively. Filtering to select only the non-redundant bi-allelic markers that are present in the parent(s) with less than 10% of missing data in the population produced 5,835 markers commonly found between the two SNP calling tools. Allelic dosage test by χ2 test showed 1,726 (29.6%) as SD markers that were used for linkage mapping.

### Linkage Map Construction

A total of 1,948 SD (SNP and SSR and eSSR) markers were used for construction of a linkage map. A framework map was built for both parental clones and the progeny using pseudo-test cross strategy (**Supplementary Figure S1**). A total of 1,437 SD markers were assigned to 294 linkage groups (LGs) with the genome coverage of 19,464 cM (**Supplementary Data Sheet S2**). Of the 294 linkage groups, 120 LGs were assigned to the maternal parent LCP 85-384 with a total map length of 4,160 cM by 378 SD markers, and 138 LGs were assigned to the paternal clone L 99-226 with genome coverage of 4,745 cM by 424 markers (**Supplementary Figure S1** and **Supplementary Data Sheet S2**). Sixty-nine LGs contained SD markers that came from both parents (D1 or D2 = 1:1). Thirty-three LGs were constructed with only SD markers that were present in both parents and segregated 3:1 (c8 or B3.7 = 3.1; **Supplementary Figure S1** and **Supplementary Data Sheet S2**). The length of the LGs varied from 0.0001 cM (LG-272) to 491 cM (LG-20) with an average of 66.20 cM per LG and an average distance of 17.03 cM between two adjacent markers. The number of mapped markers per LG varied from 2 to 31 with an average marker density of 4.89.

Homology groups (HGs) were assembled based on the mapping position of the markers in a LG on sorghum chromosomes. Of the 1,437 mapped markers in LGs, 1,027 markers (71.5%) aligned with sorghum chromosomes. Based on the synteny, 907 markers from 208 (out of 294) were grouped into 10 sorghum chromosomes and named as HG1, HG2….HG10. These 10 HGs covered 12,260 cM of the total map length, which accounted to 63% of the total genome coverage. The number of LGs grouped in a HG ranged from 5 (HG8 with 22 markers and 238.2 cM coverage) to 49 (HG1 with 272 markers and 3891 cM coverage) (**Supplementary Data Sheet S2**).

### QTL Mapping

**FIGURE 2:**
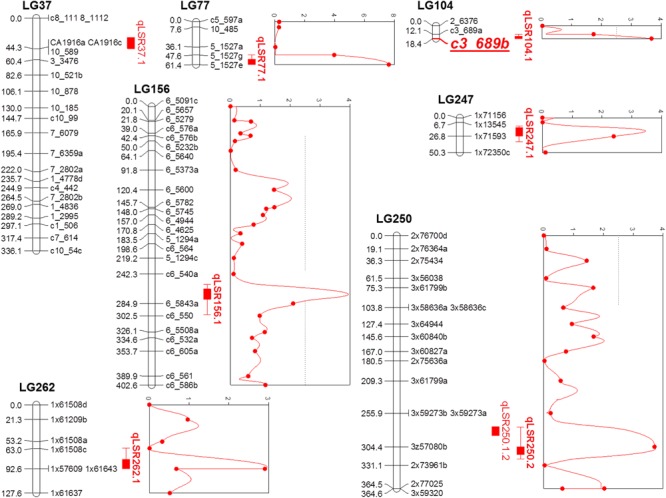
Quantitative trait locus (QTL) map using F_1_ progeny of sugarcane cultivars LCP 85-384 and L 99-226 after the saturation process of the QTL regions detected in the initial screening. For the addition of new markers, a Bonferroni correction was used in the χ^2^ test for the detection of single dose markers neighboring the QTL regions (based on the *Sorghum bicolor* information). QTLs identified using composite interval mapping are presented as red solid vertical bars with peaks on the right of linkage groups.

Composite interval mapping was performed on the quantitative phenotypic data of leaf scald reaction obtained through visual symptom severity rating using initially only the SD markers that mapped onto the linkage groups. A putative QTL was called positive when the LOD score was higher than 2.5 and the percentage of the PVE was higher than 5%. CIM identified eight QTLs on seven LGs associated with resistance to leaf scald (**Table 2** and **Figure 2**). Of these, six QTLs were identified from the mean visual data over three ratings, while one each was identified with the 2015 first and second rating data. The percentage PVE by an individual QTL for mean rating varied from 5.2 (LG 262) to 12.8 (LG 77) with 15 and 11% additive variance contributed by the resistant parent, LCP 85-384. QTLs with high additive phenotypic variance, such as qLSR37.1 (27.8%) and qLSR77.1 (54.1%) were contributed by alleles from the resistant parent. The QTL identified on LG 250 for 2015 second season explained for the highest population phenotypic variance (16.9%). Interestingly, this QTL with highest additive variation was contributed by the alleles from the susceptible parent, L 99-226. The QTL regions of six LGs were saturated with SNPs of different dosages that mapped to the sorghum genome and were not included for construction of the reference linkage map. The saturation process focused on QTL regions controlling the leaf scald response allowed for a reduction in the gap between the markers flanking some of the QTLs. Also, the recombinant LG 37 (336.09 cM), LG 104 (18.40 cM), and LG 250 (364.63 cM), which were formed after saturation with 21, 3, and 18 markers, respectively, contained one marker and two QTLs associated with leaf scald resistance (**Table 2** and **Supplementary Table S1**).

**Table 2:** QTLs associated with leaf scald resistance in the F1 progeny of LCP 85-384 × L 99-226.

QTL	Year	LG	LOD	Position (cM)	Left marker	Right marker	PVE (%)	Closest marker to peak	Add	Dom	Left CI (cM)	Right CI (cM)
qLSR37.1	2015-A	37	4.90	41.00	8_1112	CA1916a	6.69	CA1916a	–0.28	–0.07	27.85	44.25
qLSR77.1	2015-B	77	4.18	58.10	5_1527g	5_1527e	5.01	5_1527e	–0.54	0.04	50.65	61.30
qLSR77.1	Mean	77	7.63	61.30	5_1527g	5_1527e	12.83	5_1527e	–0.11	0.30	53.75	61.30
qLSR104.1	2015-A	104	2.98	17.60	c3_689a	c3_689b	2.95	c3_689b	–0.18	0.16	13.15	18.40
qLSR104.1	Mean	104	3.67	18.40	c3_689a	c3_689b	5.48	c3_689b	–0.04	0.27	14.75	18.40
qLSR156.1	2015-A	156	3.35	266.91	c6_540a	6_5843a	3.69	6_5843a	0.06	0.86	259.06	274.56
qLSR156.1	Mean	156	3.95	271.71	c6_540a	6_5843a	11.59	6_5843a	0.11	0.79	263.86	278.56
qLSR247.1	2014	247	21.90	15.70	1x13545	1x71593	1.10	1x71593	–0.10	–0.55	15.15	18.05
qLSR247.1	2015-A	247	5.26	17.70	1x13545	1x71593	3.74	1x71593	–0.04	–0.82	14.25	21.05
qLSR247.1	Mean	247	3.46	19.30	1x13545	1x71593	8.89	1x71593	0.05	–0.66	14.45	26.55
qLSR250.1	2015-B	250	3.25	281.51	3x59273a	3z57080b	16.93	3z57080b	0.76	0.17	275.06	288.06
qLSR250.2	Mean	250	3.76	306.71	3z57080b	2x73961b	7.89	3z57080b	0.24	–0.01	304.36	315.76
qLSR250.2	2015-B	250	3.13	316.41	3z57080b	2x73961b	13.59	3z57080b	0.70	0.16	313.16	319.76
qLSR262.1	2015-A	262	3.36	81.70	1x61508c	1x57609	3.95	1x57609	0.03	0.86	78.95	85.95
qLSR262.1	Mean	262	2.96	89.70	1x61508c	1x57609	5.23	1x57609	–0.15	0.12	78.75	92.65

*LG, linkage group; LOD, logarithm-base 10- of odds score (threshold = 2.5, to call a QTL positive); Position, scanning position in cM on the linkage group; PVE (%), percentage of the phenotypic variation explained by QTL at the current scanning position; Add, estimated additive effect of QTL at the current scanning position; Dom, Estimated dominance effect of QTL at the current scanning position; Left CI and Right CI, confidence intervals calculated by one-LOD drop from the estimated QTL position.*

The QTL, qLSR77.1 accounted for 12.8% of the phenotypic variation and an additive genetic variance of 0.11. To further evaluate the marker 5_1527e that was closest to the qLSR77.1 peak, allele-specific primers were run on the total 186 F1 progeny that were evaluated for leaf scald resistance. The marker, expectedly, accounted for 9% of the variation in leaf scald resistance (**Supplementary Table S2**). Based on the synteny between sugarcane and *Sorghum bicolor*, the genes located within and neighboring qLSR29.1, qLSR44.1, and qLSR77.1 were identified in sorghum. The expression of RPM1 and beta-adaptin showed up-regulation in the resistant cultivar, LCP 85-384 until 1 week after infection, whereas in susceptible cultivar, HoCP 86-845, the expression was down-regulated after an initial up-regulation at 24 h after infection (**Supplementary Table S3** and **Supplementary Figure S2**). On the other hand, the expression of PIC1 was repressed at all time points in the resistant cultivar.

## Discussion

The phenotypic resistance rating based on the severity of symptom expression in inoculated plants has been the standard method for assessment of the disease resistance response in different sugarcane clones. However, erratic symptom expression, the association between environment and symptomatology, the possibility that some sugarcane cultivars can tolerate the pathogen without exhibiting symptoms (Rott et al., 1997), and the occasional systemic infection of inoculated resistant clones (Gutierrez et al., 2016) have made leaf scald resistance evaluation a difficult task. Thus, the efficiency of marker-assisted breeding would provide a great advantage in selecting clones with resistance to leaf scald.

Visual rating of resistance based on symptom severity was used in the present study for the evaluation of the disease response in a F_1_ population progeny of a cross between a leaf scald resistant parent and a susceptible parent. The distribution of leaf scald response was skewed for broad sense heritability (H^2^) calculation (based on ANOVA test), and hence the data were transformed to obtain normal distribution. A phenotypic distribution pattern skewed toward resistance was also observed with brown rust phenotype of bi-parental progeny (Raboin et al., 2006; Yang et al., 2017). The visual symptom evaluation data could differentiate the resistant clones after data transformation (Box-Cox transformation), and the normality requirement was met for the broad sense heritability calculation by ANOVA. Using the transformed data of the visual symptom evaluation, the broad sense heritability for leaf scald resistance obtained in this study (*H*^2^ = 0.24 per plot and 0.48 per mean) was similar to other sugarcane diseases, such as red rot (*H*^2^ = 0.19 to 0.31; Yin et al., 1996) and smut (*H*^2^ = 0.41 ± 0.08; Chao et al., 1990). The low to moderate H^2^ value obtained in the present study was due to the erratic symptom expression of the disease and latency, and this outcome is not surprising given the well documented influence of the environment on symptom expression. On the other hand, very high broad sense *H*^2^ (0.98) was reported for leaf scald resistance in a different environment with crosses involving Brazilian clones by Bressiani et al. (2007). However, the authors used the first stubble, and the disease rating was done 9 months after inoculation.

Linkage mapping in sugarcane requires a large number of progeny and markers in comparison with diploid plants as the low number of markers decreases the reliability of estimating useful genetic distances between the markers (Andru et al., 2011). In the present study, the selective genotyping by NGS of a relatively small population produced a sufficiently large number of markers, which along with the use of the synteny between *S. bicolor* and *Saccharum* spp. (Wang et al., 2010; Aitken et al., 2014) allowed the construction of a reliable and informative linkage map that was comparable with previously reported sugarcane linkage maps (Aitken et al., 2014; Yang et al., 2017). SNP calling by Tassel, as used in the present study, has been shown to be better in calling SD SNPs compared to other SNP callers (Yang et al., 2017). Further, the exclusive use of SD markers for the construction of the framework linkage map, followed by the saturation process with previously unmapped SD markers ensured high reliability in estimating genetic distances (Andru et al., 2011). In sugarcane, LOD scores ≥ 3.0 and recombination frequency values between 0.25 and 0.45 have been commonly used (Da Silva et al., 1993; Grivet et al., 1996; Alwala et al., 2008; Andru et al., 2011), although the maximum detectable recombination generally depends on the size of the mapping population (Andru et al., 2011). In the present study, a maximum recombination frequency value of 0.40 and LOD score values ≥ 4.0 were used to avoid false linkages. However, the high number of unlinked markers, short LGs with less than four markers per LG, long distance between some markers, and the presence of long LGs (LG 20, for example) despite the use of LOD scores, and recombination thresholds similar to previously reported linkage map studies (Da Silva et al., 1993; Grivet et al., 1996; Alwala et al., 2008; Andru et al., 2011) were possibly due to the small population size. The inclusion of redundant variants that were removed during the framework linkage map construction helped circumvent this problem. In addition, inclusion of more SD markers for mapping, will be helpful to generate saturated LGs with higher numbers of markers. The number of LGs in the resistant parent LCP 85-384 (120) was more than 106 LGs reported earlier (Andru et al., 2011). Similarly, the number of LGs in the susceptible parent L 99-226 (137) was expectedly more than the ideally expected number 120. Such genetic map fragmentation in L99-226 could be due to a lower number of polymorphic markers due to inbreeding-caused reduced heterozygosity in modern sugarcane (Deren, 1995), and low marker density and uneven distribution of linked SD markers. In addition, the high number of recombinant linkage groups in the present study could be attributed to the small mapping population size. Since all the SNPs used in the present mapping were called based on the sorghum reference genome, all LGs were assembled into 10 homeologous groups (HG) of sorghum. This further supports the high degree of collinearity between the two crops (Ming et al., 1998). Using the physical position information of the markers in sorghum genome could be a better strategy to place the LGs into HGs with confidence instead of the conventional method where LGs sharing two or more common multi-allelic markers from a same locus were grouped into one HG (Andru et al., 2011; Aitken et al., 2014).

QTL analysis identified eight genomic regions on seven LGs controlling leaf scald response, which cumulatively explained 89% of the phenotypic variation. QTLs, qLSR37.1, qLSR77.1, qLSR104.1, and qLSR262.1, together, accounted for 30% of the resistance response where the alleles were contributed by the resistant parent. This suggested that alleles of these QTLs could be pyramided for obtaining quantitative resistance against leaf scald. In addition, single marker analysis (SMA) also identified four SD markers associated with leaf scald resistance with PVE ranging from 9.42% for marker c3_579 on LG29 to 17.27% for marker c3_689 on LG 104 (**Supplementary Table S1**). However, small number of progeny used for QTL mapping in this study could result in identification of genomic regions with overestimated phenotypic variation. Allele-specific markers specific to the SNP markers c5_1527 (12% PVE by CIM), c3_689 (17% PVE by SMA), and c3_579 (9% PVE by SMA) were run on the complete set of 186 progeny from the population, and the regression analysis showed that the three markers contributed 9, 4, and 3% of the phenotypic variation, respectively (**Supplementary Table S2**). Using syntenic information of the *S. bicolor* genome, one representative gene was selected around three QTLs (**Supplementary Table S3**). The induction/repression of expression of the genes in leaf/meristem tissue implicated their role in leaf scald resistance in sugarcane.

The QTL flanked by 5_1527g and 5_1527e (LG 77, 12.8% PVE) served as the starting point for subsequent analysis because of the high value of PVE and the information on the expression of the neighboring ESTs/genes that are associated with disease resistance. Pinpointing causative genes/markers within/around QTLs suggested that the QTL analysis and the use of the microsynteny between *S. bicolor* and *Saccharum* spp. could be a valuable tool in sugarcane research. Subsequent analysis of allelic polymorphism and comprehensive gene expression profile around the QTLs can enhance our knowledge of the nature of leaf scald resistance in sugarcane. These results further suggested that other QTLs identified in the present study need to be fine mapped to identify diagnostic SNPs linked to leaf scald resistance.

The GBS-derived SNP-enriched genetic map developed in the present study coupled with comparative analysis with the sorghum genome overcame the limitations associated with the small population used in the mapping process and the high environmental influence in the symptom expression of the disease, in addition to providing improved understanding of the sugarcane genome structure. Marker c5_1527 tightly linked to qLSR77, being a codominant, could be used, in combination with other linked SNPs, as leaf scald resistance diagnostic markers in marker-assisted breeding. Validation of the markers identified in this study is being performed using diverse germplasm with known leaf scald reaction. The validated molecular markers linked to leaf scald resistance can be used as new selection tools for large-scale screening of parents and early generation progeny in the breeding program to develop resistant cultivars.

## Author Contributions

NB and JH conceptualized the study, designed the experiment, and revised the manuscript. CK provided the materials. AG, JH, and NB performed the experiment. AG analyzed the data and wrote the first draft of the manuscript. All authors read and approved the manuscript.

## Conflict of Interest Statement

The authors declare that the research was conducted in the absence of any commercial or financial relationships that could be construed as a potential conflict of interest.

## Supplementary Material

FIGURE S1 Framework linkage map of F_1_ progeny of sugarcane cultivars LCP 85-384 and L 99-226 constructed using single dose SSR and SNP markers.

FIGURE S2 Quantitative real-time PCR showing temporal changes in the expression of one selected gene from three QTLs in the leaf/meristematic tissues of a leaf scald resistant clone (LCP 85-384) and susceptible clone (HoCP 85-845). RNA was isolated from meristematic tissues of three independent plants (biological replicates) at 0 h (control), 24 h, 48 h, 72 h and 1 week after leaf scald inoculation. First strand synthesis, real-time PCR, and fold-change relative expression was performed as described earlier (Baisakh et al., 2012).

TABLE S1 Single dose markers associated with the leaf scald response in the LCP85-384 × L 99-226 F1 population as identified by single marker analysis.

TABLE S2 Regression analysis showing contribution of individual SNP markers associated with leaf scald resistance on 186 progeny of the LCP85-384 × L 99-226 F1 population.

TABLE S3 Genes known to be involved in biotic stress response, which are close to the QTLs identified, and used for expression analysis.

DATA SHEET S1 ANOVA for heritability of leaf scald reaction of sugarcane clones under field conditions.

DATA SHEET S2 Linkage mapping of the F1 progeny of LCP 85-384 and L 99-226 showing linkage groups (LGs) and homology groups (HGs).

## References

[B1] Aitken K. S. Jackson P. A. McIntyre C. L. (2006). Quantitative trait loci identified for sugar related traits in sugarcane (*Saccharum* spp.) cultivar x *Saccharum officinarum* population. *Theor. Appl. Genet.* 112 1306–1317. 10.1007/s00122-006-0233-2 16508765

[B2] Aitken K. S. McNeil M. D. Hermann S. Bundock P. C. Kilian A. Heller-Uszynska K. (2014). A comprehensive genetic map of sugarcane that provides enhanced map coverage and integrates high-throughput Diversity Array Technology (DArT) markers. *BMC Genomics* 15:152. 10.1186/1471-2164-15-152 24564784 PMC4007999

[B3] Aljanabi S. M. Parmessur Y. Kross H. Dhayan S. Saumtally S. Ramdoyal K. (2007). Identification of a major quantitative trait locus (QTL) for yellow spot (*Mycovellosiella koepkei*) disease resistance in sugarcane. *Mol. Breed.* 19 1–14. 10.1007/s11032-006-9008-3

[B4] Alwala S. Kimbeng C. A. Veremis J. C. Gravois K. A. (2008). Linkage mapping and genome analysis in *Saccharum* interspecific cross using AFLP, SRAP and TRAP markers. *Euphytica* 164 37–51. 10.1007/s10681-007-9634-9

[B5] Andru S. Pan Y. B. Thongthawee S. Burner D. M. Kimbeng C. A. (2011). Genetic analysis of the sugarcane (*Saccharum* spp.) cultivar ‘LCP 85-384’. I. Linkage mapping using AFLP, SSR, and TRAP markers. *Theor. Appl. Genet.* 123 77–93. 10.1007/s00122-011-1568-x 21472411

[B6] Asnaghi C. Roques D. Ruffel S. Kaye C. Hoarau J. Y. Télismart H. (2004). Targeted mapping of a sugarcane rust resistance gene (Bru1) using bulked segregant analysis and AFLP markers. *Theor. Appl. Genet.* 108 759–764. 10.1007/s00122-003-1487-6 14586507

[B7] Baer O. T. Lalusin A. G. (2013). Molecular markers associated to downy mildew [*Peronosclerospora philippinensis* (W. Weston) C.G. Shaw] resistance in sugarcane (*Saccharum officinarum* L.) hybrids (CP 57-604 × PHIL 84-77). *Phlipp. J. Crop Sci.* 38 37–45.

[B8] Baisakh N. RamanaRao M. V. Rajasekaran K. Subudhi P. Galbraith D. Jonda J. (2012). Enhanced salt stress tolerance of rice plants expressing a vacuolar H+-ATPase subunit c1 (SaVHAc1) gene from a halophyte grass *Spartina alterniflora* Löisel. *Plant Biotechnol. J.* 10 453–464. 10.1111/j.1467-7652.2012.00678.x 22284568

[B9] Balsalobre T. W. A. Da Silva Pereira G. Margarido G. R. A. Gazaffi R. Barreto F. Z. Anoni C. O. (2017). GBS-based single dosage markers for linkage and QTL mapping allow gene mining for yield-related traits in sugarcane. *BMC Genomics* 18:72. 10.1186/s12864-016-3383-x 28077090 PMC5225503

[B10] Bischoff K. P. Gravois K. A. Reagan T. E. Hoy J. W. Laborde C. M. Kimbeng C. A. (2009). Registration of ‘L 99-226’ Sugarcane. *J. Plant Regist.* 3 241–247. 10.3198/jpr2009.04.0210crc

[B11] Bressiani J. A. Burnquist W. Da Silva J. A. (2007). Breeding sugarcane for leaf scald resistance: a genetic study. *J. Am. Soc. Sugar Cane Technol.* 27 15–22.

[B12] Chao C. P. Hoy J. W. Saxton A. M. Martin F. A. (1990). Heritability of resistance of clone reaction to sugarcane smut in Louisiana. *Phytopathology* 80 622–626. 10.1094/Phyto-80-622

[B13] Churchill G. A. Doerge R. W. (1994). Empirical threshold values for quantitative trait mapping. *Genetics* 138 963–971. 7851788 10.1093/genetics/138.3.963 PMC1206241

[B14] Cordeiro G. M. Taylor G. O. Henry R. J. (2000). Characterization of microsatellite markers from sugarcane (*Saccharum* sp.), a highly polymorphic species. *Plant Sci.* 155 161–168. 10.1016/S0168-9452(00)00208-9 10814819

[B15] Costet L. Cunff L. Royaert S. Raboin L. M. Hervouet C. Toubi L. (2012a). Haplotype structure around Bru1 reveals a narrow genetic basis for brown rust resistance in modern sugarcane cultivars. *Theor. Appl. Genet.* 125 825–836. 10.1007/s00122-012-1875-x 22572763

[B16] Costet L. Raboin L. M. Payet M. D’Hont A. Nibouche S. (2012b). A major quantitative trait allele for resistance to the *Sugarcane yellow leaf virus (Luteoviridae)*. *Plant Breed.* 131 637–640. 10.1111/j.1439-0523.2012.02003.x

[B17] Da Silva J. A. G. Sorrells M. E. Burnquist W. L. Tanksley S. D. (1993). RFLP linkage map and genome analysis of *Saccharum spontaneum*. *Genome* 36 782–791. 10.1139/g93-103 18470024

[B18] Daugrois J. H. Grivet L. Roques D. Hoarau J. Y. Lombard H. Glaszmann J. C. (1996). Putative major gene for rust resistance linked with a RFLP marker in sugarcane cultivar ‘R570’. *Theor. Appl. Genet.* 92 1059–1064. 10.1007/BF00224049 24166636

[B19] Debibakas S. Rocher S. Garsmeur O. Toubi L. Roques D. D’Hont A. (2014). Prospecting sugarcane resistance to sugarcane yellow leaf virus by genome-wide association. *Theor. Appl. Genet.* 127 1719–1732. 10.1007/s00122-014-2334-7 24916990 PMC4110414

[B20] Dellaporta S. L. Wood J. Hicks J. B. (1983). A plant DNA minipreparation: version II. *Plant Mol. Biol. Rep.* 1 19–21. 10.1007/BF02712670

[B21] Deren C. W. (1995). Genetic base of U.S. mainland sugarcane. *Crop Sci.* 35 1195–1199. 10.2135/cropsci1995.0011183X003500040047x 26214470

[B22] D’Hont A. Glaszmann J. C. (2001). Sugarcane genome analysis with molecular markers: a first decade or research. *Proc. Int. Soc. Technol.* 24 556–559.

[B23] D’Hont A. Grivet L. Feldmann P. Rao S. Berding N. Glaszmann J. C. (1996). Characterization of the double genome structure of modern sugarcane cultivars (*Saccharum* spp.) by molecular cytogenetics. *Mol. Gen. Genet.* 250 405–413. 10.1007/BF02174028 8602157

[B24] D’Hont A. Ison D. Alix K. Roux C. Glazmann J. C. (1998). Determination of basic chromosome numbers in the genus *Saccharum* by physical mapping of ribosomal RNA genes. *Genome* 41 221–225. 10.1139/g98-023

[B25] Drenkard E. Richter B. G. Rozen S. Stutius L. M. Angell N. A. Mindrinos M. (2000). A simple procedure for the analysis of single nucleotide polymorphisms facilitates map-based cloning in Arabidopsis. *Plant Physiol.* 124 1483–1492. 10.1104/pp.124.4.1483 11115864 PMC1539302

[B26] Elshire R. J. Glaubitz J. C. Sun Q. Poland J. A. Kawamoto K. Buckler E. S. (2011). A robust, simple genotyping-by-sequencing (GBS) approach for high diversity species. *PLoS One* 6:e19379. 10.1371/journal.pone.0019379 21573248 PMC3087801

[B27] Garces F. F. Gutierrez A. Hoy J. W. (2014). Detection and quantification of *Xanthomonas albilineans* by qPCR and potential characterization of sugarcane resistance to leaf scald. *Plant Dis.* 98 121–126. 10.1094/PDIS-04-13-0431-RE 30708616

[B28] Glaubitz J. C. Casstevens T. M. Lu F. Harriman J. Elshire R. J. Sun Q. (2014). TASSEL-GBS: a high capacity genotyping by sequencing analysis pipeline. *PLoS One* 9:e90346. 10.1371/journal.pone.0090346 24587335 PMC3938676

[B29] Glynn N. C. Laborde C. Davidson R. W. Irey M. S. Glaz B. DHont A. (2013). Utilization of a major brown rust resistance gene in sugarcane breeding. *Mol. Breed.* 31 323–331. 10.1007/s11032-012-9792-x 28821913

[B30] Grattapaglia D. Sederoff R. (1994). Genetic linkage maps of *Eucalyptus grandis* and *Eucalyptus urophylla* using a pseudo-testcross: mapping strategy and RAPD markers. *Genetics* 137 1121–1137. 7982566 10.1093/genetics/137.4.1121 PMC1206059

[B31] Grivet L. D’Hont A. Roques D. Feldmann P. Lanaud C. Glaszmann J. C. (1996). RFLP mapping in cultivated sugarcane (*Saccharum* spp.): genome organization in a highly polyploid and aneuploid interspecific hybrid. *Genetics* 142 987–1000. 8849904 10.1093/genetics/142.3.987 PMC1207035

[B32] Guimaraes C. T. Honeycutt R. J. Sills G. R. Sobral B. W. S. (1999). Genetic linkage maps of *Saccharum officinarum* L. and *Saccharum robustum* Brandes & Jew. Ex grassl. *Genet. Mol. Biol.* 22 125–132. 10.1590/S1415-47571999000100024

[B33] Gutierrez A. F. Garces F. F. Hoy J. W. (2016). Evaluation of resistance to leaf scald by quantitative PCR of *Xanthomonas albilineans* in sugarcane. *Plant Dis.* 100 1331–1338. 10.1094/PDIS-09-15-1111-RE 30686195

[B34] Hoarau J. Y. Offmann B. D’Hont A. Risterucci A. M. Roques D. Glaszmann J. C. (2001). Genetic dissection of a modern sugarcane cultivar (*Saccharum* spp.). I. Genome mapping with AFLP markers. *Theor. Appl. Genet.* 103 84–97. 10.1007/s001220000390 12582930

[B35] Khan N. A. Bedre R. Parco A. Bernaola L. Hale A. Kimbeng C. (2013). Identification of cold-responsive genes in energycane for their use in genetic diversity analysis and future functional marker development. *Plant Sci.* 211 122–131. 10.1016/j.plantsci.2013.07.001 23987817

[B36] Koike H. (1965). Aluminum-cap method for testing sugarcane varieties against leaf scald disease. *Phytopathology* 55 317–319.

[B37] Le Cunff L. Garsmeur O. Raboin L. M. Pauquet J. Telismart H. Selvi A. (2008). Diploid/polyploid syntenic shuttle mapping and haplotype specific chromosome walking toward a rust resistance gene (Bru1) in highly polyploid sugarcane (2n-12x-115). *Genetics* 180 649–660. 10.1534/genetics.108.091355 18757946 PMC2535714

[B38] Li H. Handsaker B. Wysoker A. Fennell T. Ruan J. Homer N. (2009). The sequence alignment/Map format and SAMtools. *Bioinformatics* 25 2078–2079. 10.1093/bioinformatics/btp352 19505943 PMC2723002

[B39] Margarido G. R. A. Souza A. P. Garcia A. A. F. (2007). OneMap: software for genetic mapping in outcrossing species. *Hereditas* 144 78–79. 10.1111/j.2007.0018-0661.02000.x 17663699

[B40] Milligan S. B. Martin F. A. Bischoff K. P. Quebedeaux J. P. Dufrene E. O. Quebedeaux K. L. (1994). Registration of ‘LCP 85-384’ sugarcane. *Crop Sci.* 34 819–820. 10.2135/cropsci1994.0011183X003400030042x

[B41] Ming R. Liu S. C. Lin Y. R. Da Silva J. A. G. Wilson W. Braga D. (1998). Detailed alignment of *Saccharum* and *Sorghum* chromosomes: comparative organization of closely related diploid and polyploid genomes. *Genetics* 150 1663–1682. 9832541 10.1093/genetics/150.4.1663 PMC1460436

[B42] Pan Y.-B. (2006). Highly polymorphic microsatellite DNA markers for sugarcane germplasm evaluation and variety identity testing. *Sugar Technol.* 8 246–256. 10.1007/BF02943564

[B43] Parco A. S. Avellaneda M. C. Hale A. H. Hoy J. W. Kimbeng C. A. Pontif M. J. (2014). Frequency and distribution of the brown rust resistance gene *Bru*1 and implications for the Louisiana sugarcane breeding programme. *Plant Breed.* 133 654–659. 10.1111/pbr.12186

[B44] Parco A. S. Hale A. H. Avellaneda M. C. Hoy J. W. Kimbeng C. A. Pontif M. J. (2017). Distribution and frequency of Bru 1, a major brown rust resistance gene, in the sugarcane world collection. *Plant Breed.* 136 637–651. 10.1111/pbr.12508

[B45] Raboin L.-M. Oliveira K. M. Lecunff L. Telismart H. Roques D. Butterfield M. (2006). Genetic mapping in sugarcane, a high polyploid, using bi-parental progeny: identification of a gene controlling stalk colour and a new rust resistance gene. *Theor. Appl. Genet.* 112 1382–1391. 10.1007/s00122-006-0240-3 16552554

[B46] Racedo J. Perera M. F. Bertani R. Funes C. Gonzales V. Cuenya M. I. (2013). Bru1 gene and potential alternative sources of resistance to sugarcane brown rust disease. *Euphytica* 191 429–436. 10.1007/s10681-013-0905-3

[B47] Ricaud C. Ryan C. C. (1989). “Leaf scald,” in *Diseases of Sugarcane. Major Diseases* eds Ricaud C. Egan B. T. Gillaspie A. G. Jr. Hughes C. G. (Amsterdam: Elsevier Science Publishers B.V) 39–58. 10.1016/B978-0-444-42797-7.50007-7

[B48] Rossi M. Araujo P. G. Paulet F. Garsmeur O. Dias V. M. Chen H. (2003). Genomic distribution and characterization of EST-derived resistance gene analogs (RGAs) in sugarcane. *Mol. Gen. Genomics* 269 406–419. 10.1007/s00438-003-0849-8 12733061

[B49] Rott P. Davis M. J. (2000). “Leaf scald,” in *A Guide to Sugarcane Diseases* eds Rott P. Bailey R. A. Comstock J. C. Croft B. J. Saumtally A. S. (Montpellier: CIRAD-ISSCT) 38–44.

[B50] Rott P. Mohamed I. S. Klett P. Soupa D. de Saint-Albin A. Feldmann P. (1997). Resistance to leaf scald disease is associated with limited colonization of sugarcane and wild relatives by *Xanthomonas albilineans*. *Phytopathology* 87 1202–1213. 10.1094/PHYTO.1997.87.12.1202 18945019

[B51] Singh R. K. Singh S. P. Tiwari D. K. Srivastava S. Singh S. B. Sharma M. L. (2013). Genetic mapping and QTL analysis for sugar yield-related traits in sugarcane. *Euphytica* 191 333–353. 10.1007/s10681-012-0841-7 27342657

[B52] Solis J. Gutierrez A. Mangu V. Sanchez E. Bedre R. Linscombe S. Baisakh N. (2017). Genetic mapping of quantitative trait loci for grain yield under drought in rice under controlled greenhouse conditions. *Front. Chem.* 5: 129. 10.3389/fchem.2017.00129 29359127 PMC5766644

[B53] Voorrips R. E. (2002). MapChart: software for the graphical presentation of linkage maps and QTLs. *J. Hered.* 93 77–78. 10.1093/jhered/93.1.77 12011185

[B54] Wang J. Li H. Zhang L. Meng L. (2016). *Users’ Manual of QTL IciMapping v3.1.* Beijing: Institute of Crop Science, Chinese Academy of Agricultural Sciences (CAAS).

[B55] Wang J. Roe B. Macmil S. Yu Q. Murray J. Tang H. (2010). Microcollinearity between autopolyploid sugarcane and diploid sorghum genomes. *BMC Genomics* 11:261. 10.1186/1471-2164-11-261 20416060 PMC2882929

[B56] Wang S. Basten C. Zeng Z. (2012). *Windows QTL Cartographer 2.5.* Raleigh, NC: North Carolina State University.

[B57] Wang Z. K. Comstock J. C. Hatziloukas E. Schaad N. W. (1999). Comparison of PCR, BIO-PCR, DIA, ELISA and isolation on semiselective medium for detection of *Xanthomonas albilineans*, the causal agent of leaf scald of sugarcane. *Plant Pathol.* 48 245–252. 10.1046/j.1365-3059.1999.00332.x

[B58] Wu K. K. Burnquist W. Sorrells M. E. Tew T. L. Moore P. H. Tanksley S. D. (1992). The detection and estimation of linkage in polyploids using single-dose restriction fragments. *Theor. Appl. Genet.* 83 294–300. 10.1007/BF00224274 24202510

[B59] Wu R. Ma C. X. Painter I. Zeng Z. B. (2002). Simultaneous maximum likelihood estimation of linkage and linkage phases in outcrossing species. *Theor. Popul. Biol.* 61 349–363. 10.1006/tpbi.2002.1577 12027621

[B60] Yang X. Song J. You Q. Paudel D. R. Zhang J. Wang J. (2017). Mining sequence variations in representative polyploid sugarcane germplasm accessions. *BMC Genomics* 18:594. 10.1186/s12864-017-3980-3 28793856 PMC5551020

[B61] Yin Z. Hoy J. W. Milligan S. B. (1996). Evaluation and heritability of resistance to sugarcane red rot. *Phytopathology* 86 662–667. 10.1094/Phyto-86-662

