# Supplementary material for: Identification of Genomic Regions Controlling Leaf Scald Resistance in Sugarcane Using a Bi-parental Mapping Population and Selective Genotyping by Sequencing
Source: Front Plant Sci. 2018 Jun 26;9:877. doi: 10.3389/fpls.2018.00877 (PMC6028728; doi:10.3389/fpls.2018.00877)
Supplement: FIGURE S1 — Framework linkage map of F1 progeny of sugarcane cultivars LCP 85-384 and L 99-226 constructed using single dose SSR and SNP markers. [file Presentation_1.PPTX]

## Slide 1
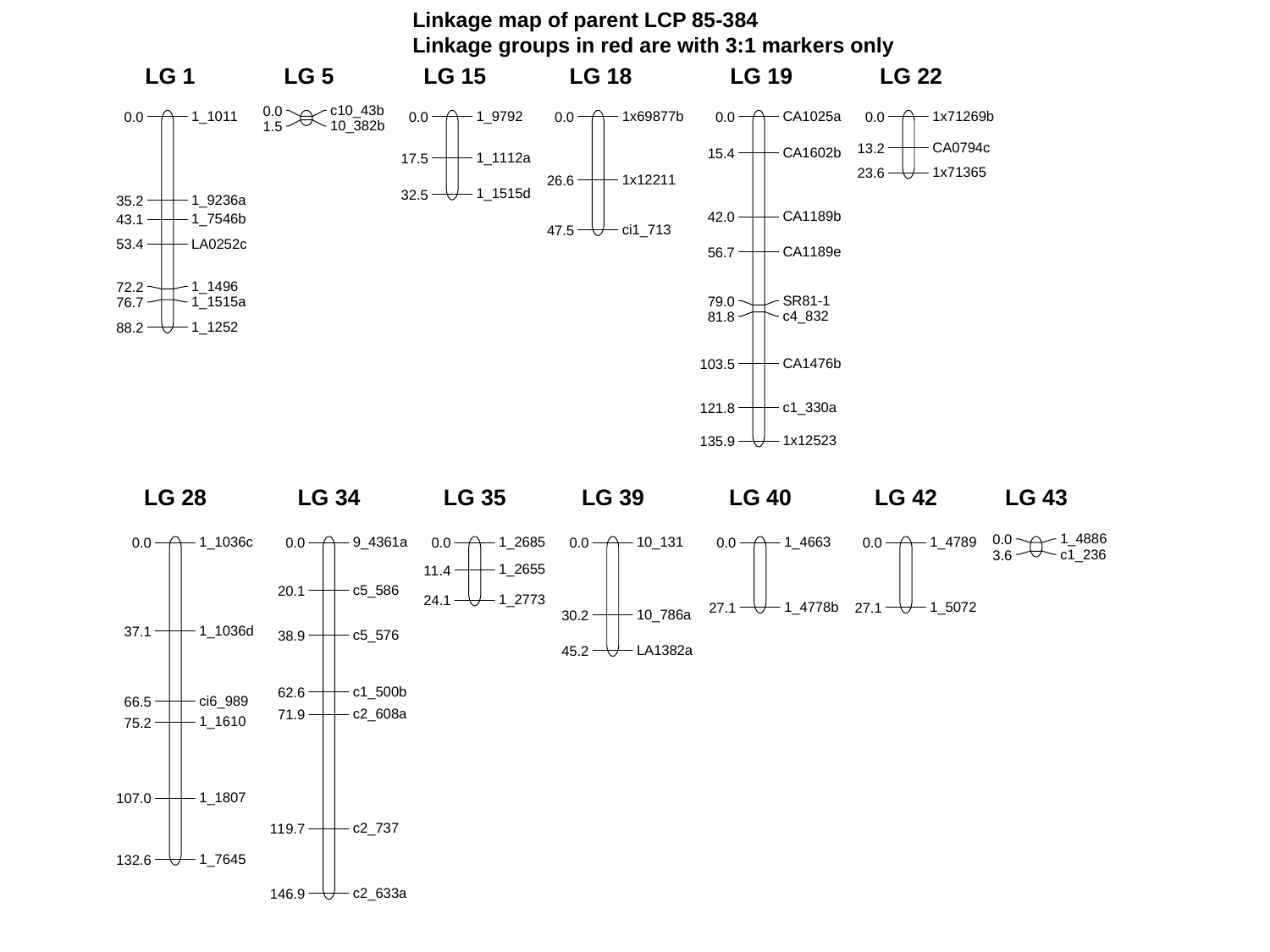

Linkage map of parent LCP 85-384
Linkage groups in red are with 3:1 markers only

## Slide 2
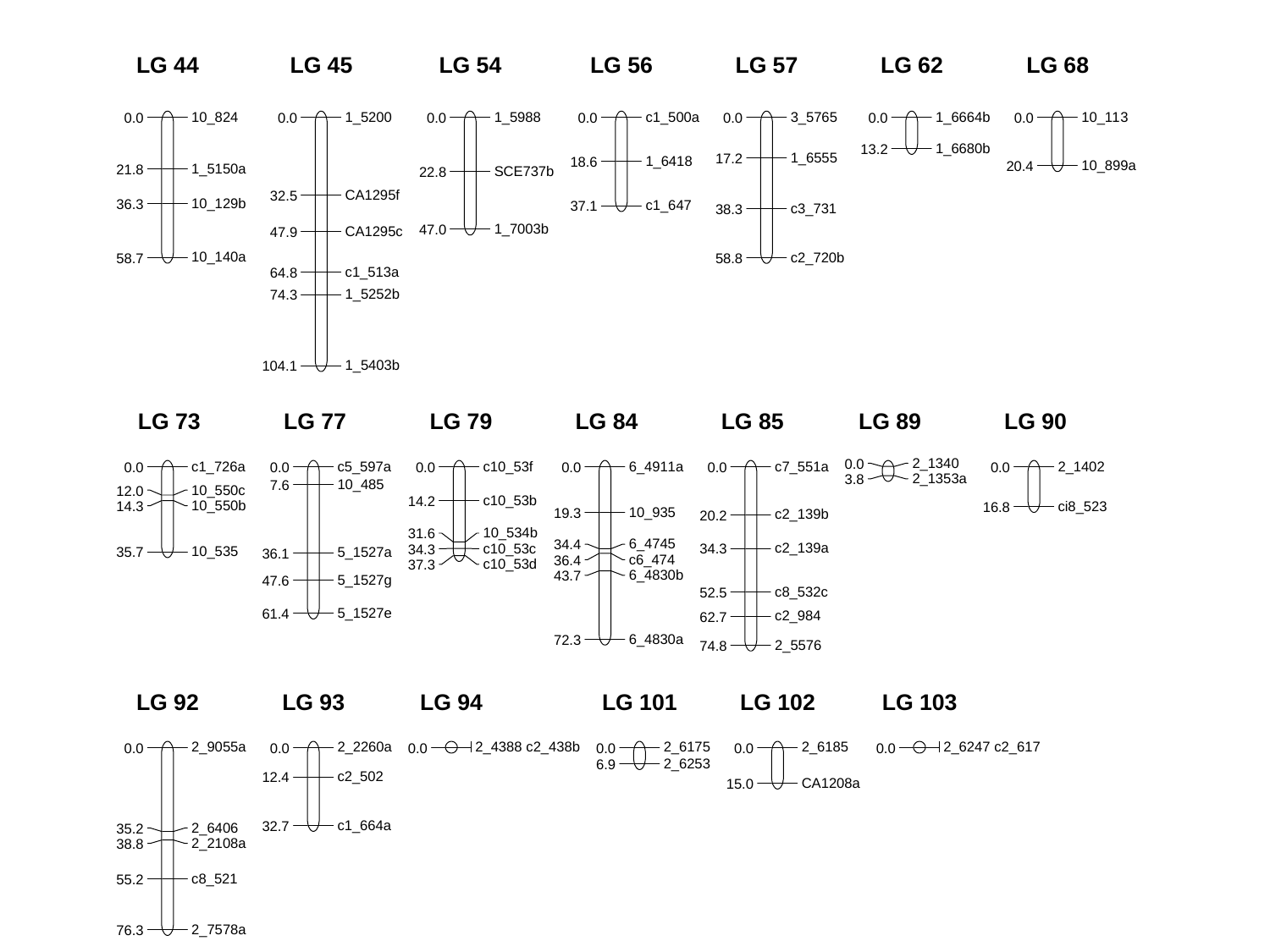

## Slide 3
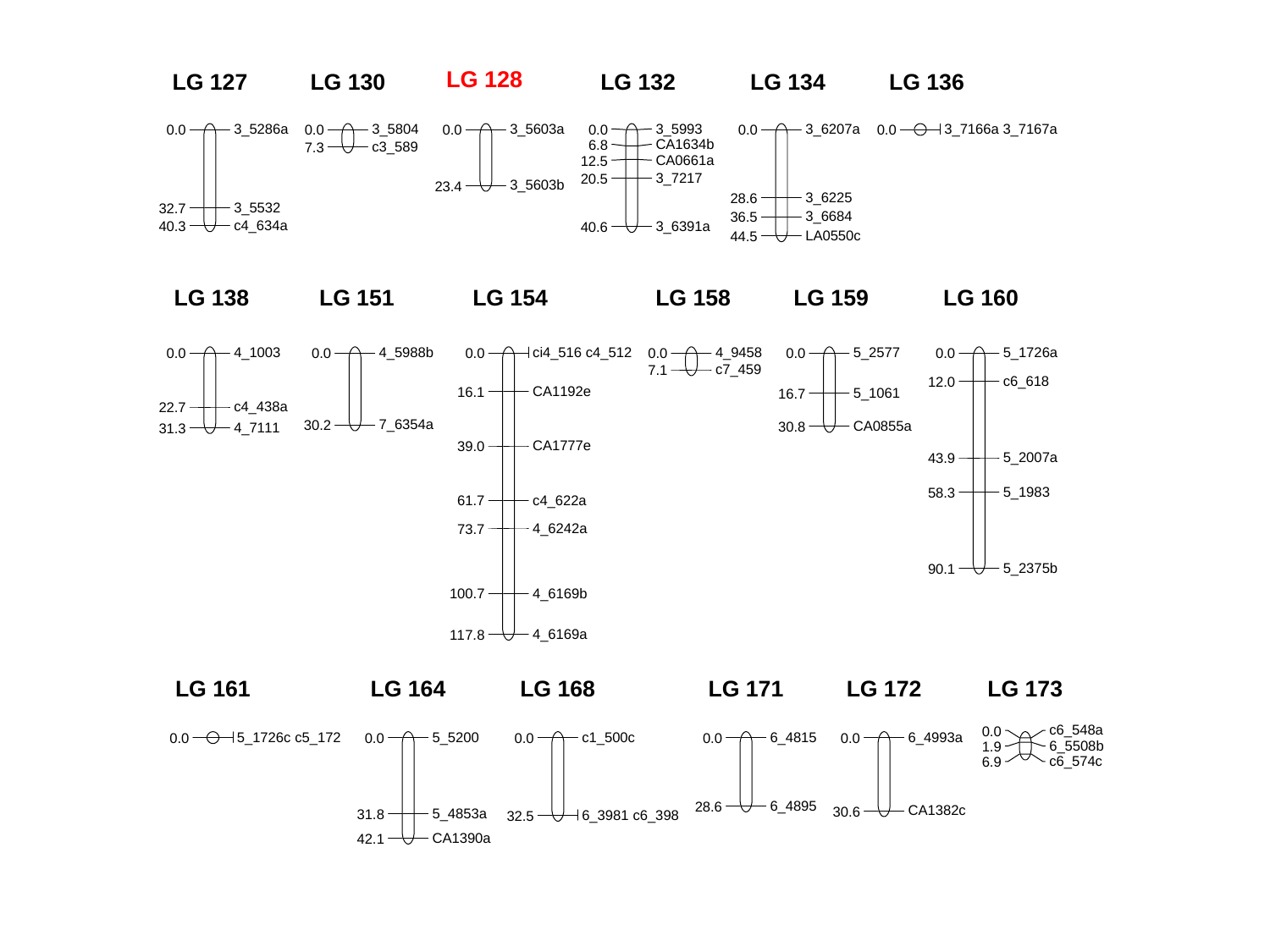

LG 128

## Slide 4
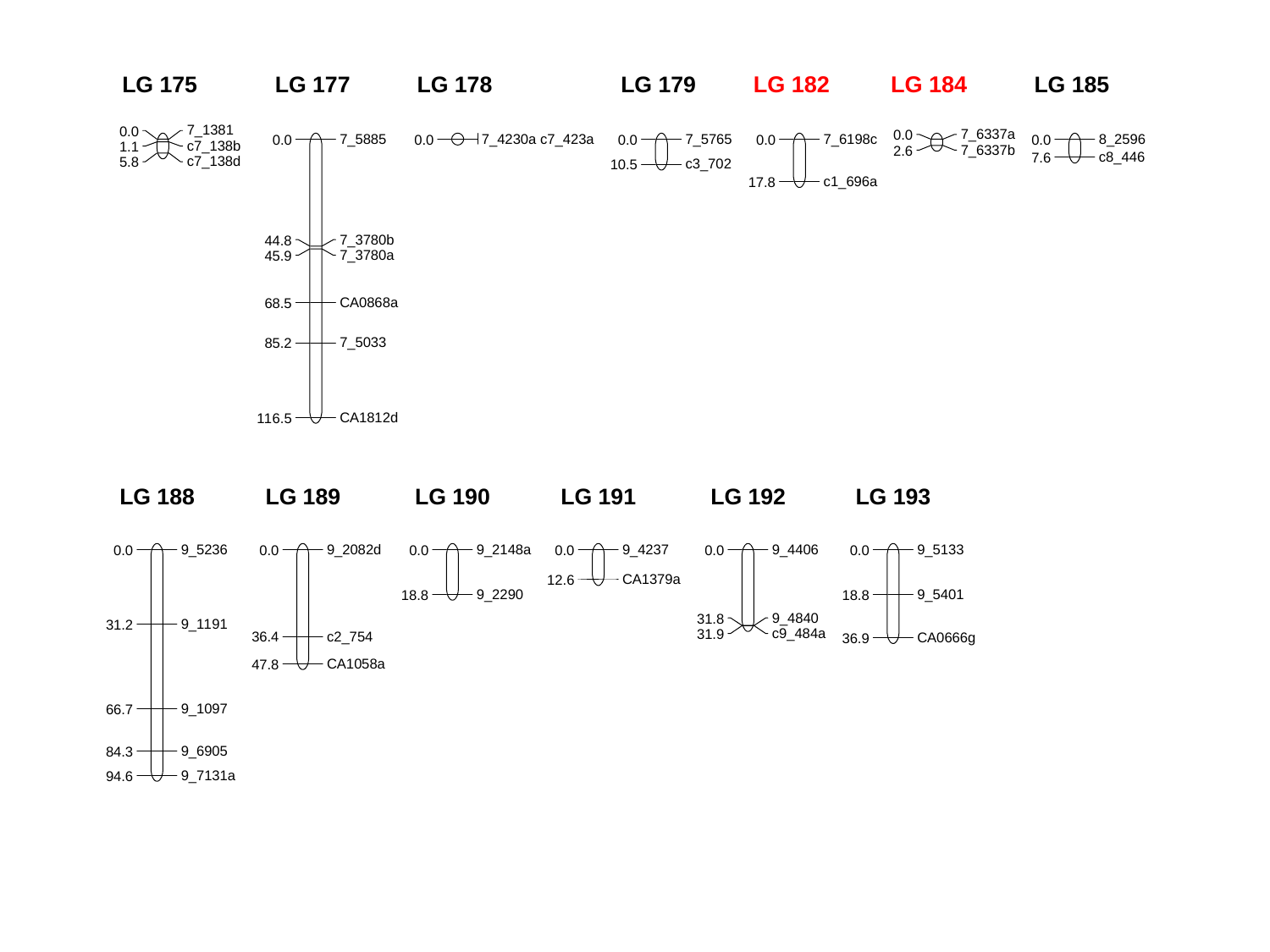

LG 182
LG 184

## Slide 5
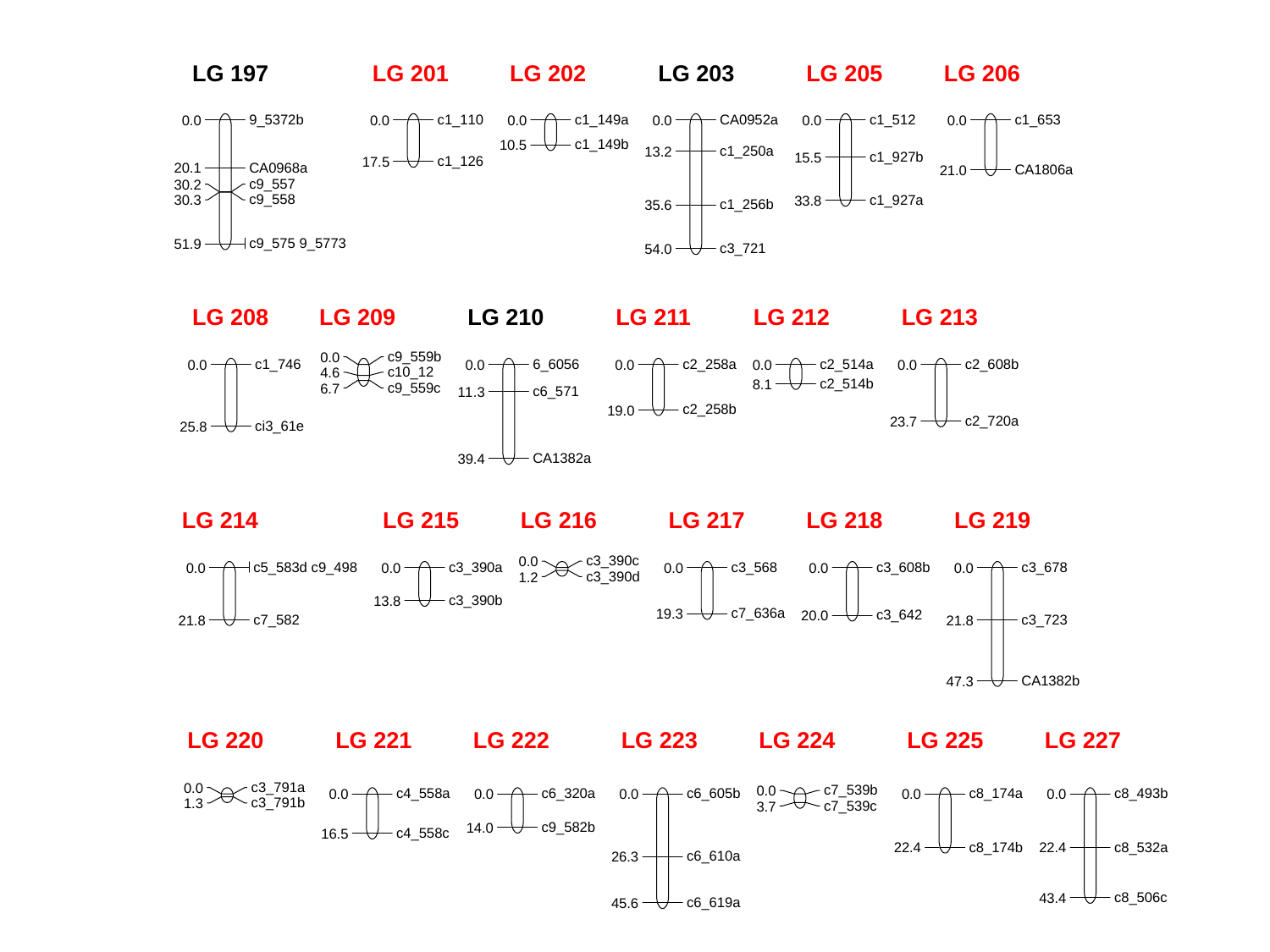

LG 197
LG 201
LG 202
LG 203
LG 205
LG 206
LG 208
LG 209
LG 210
LG 211
LG 212
LG 213
LG 214
LG 215
LG 216
LG 217
LG 218
LG 219
LG 220
LG 221
LG 222
LG 223
LG 224
LG 225
LG 227

## Slide 6
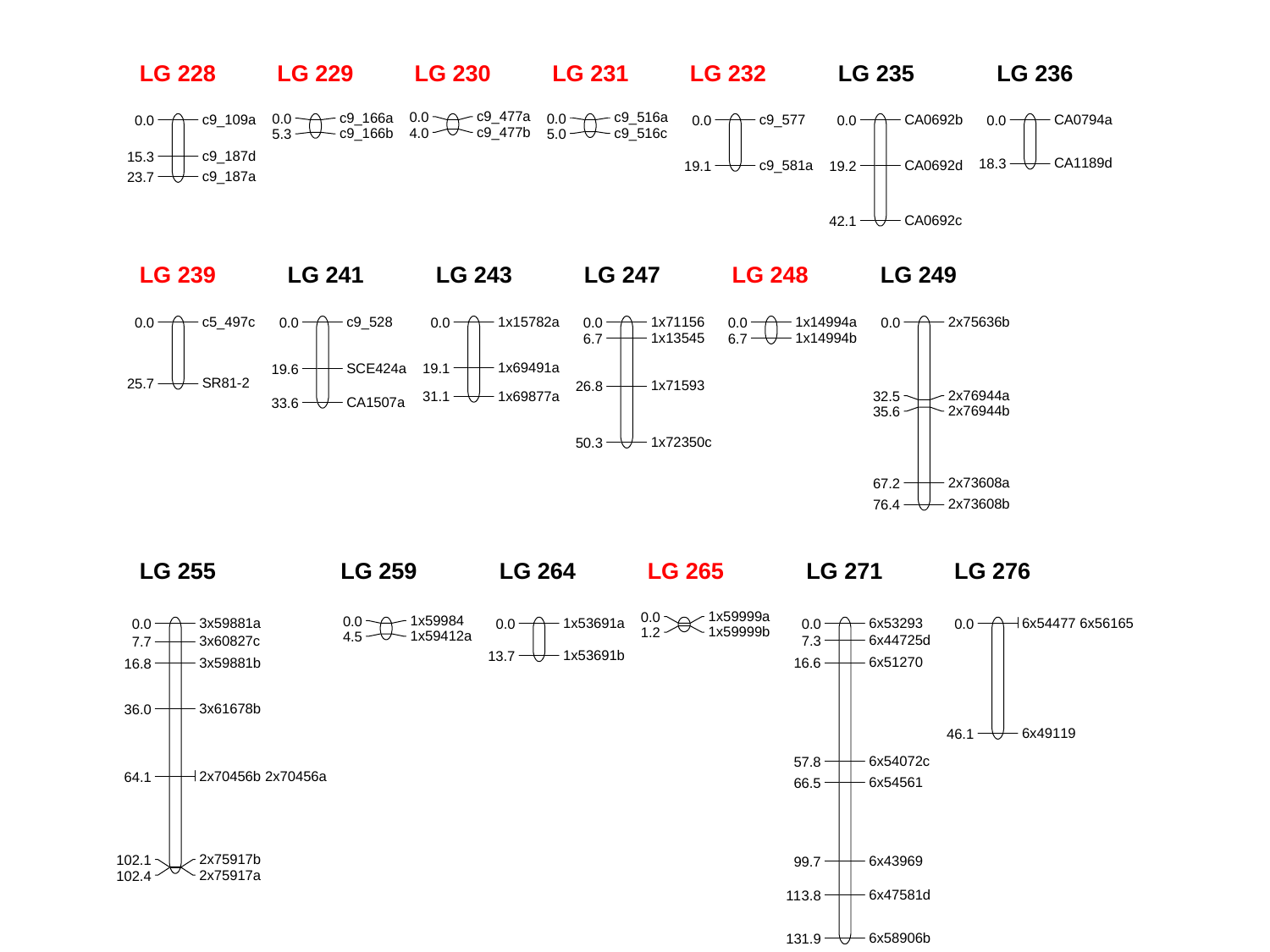

LG 228
LG 229
LG 230
LG 231
LG 232
LG 235
LG 236
LG 239
LG 241
LG 243
LG 247
LG 248
LG 249
LG 255
LG 259
LG 264
LG 265
LG 271
LG 276

## Slide 7
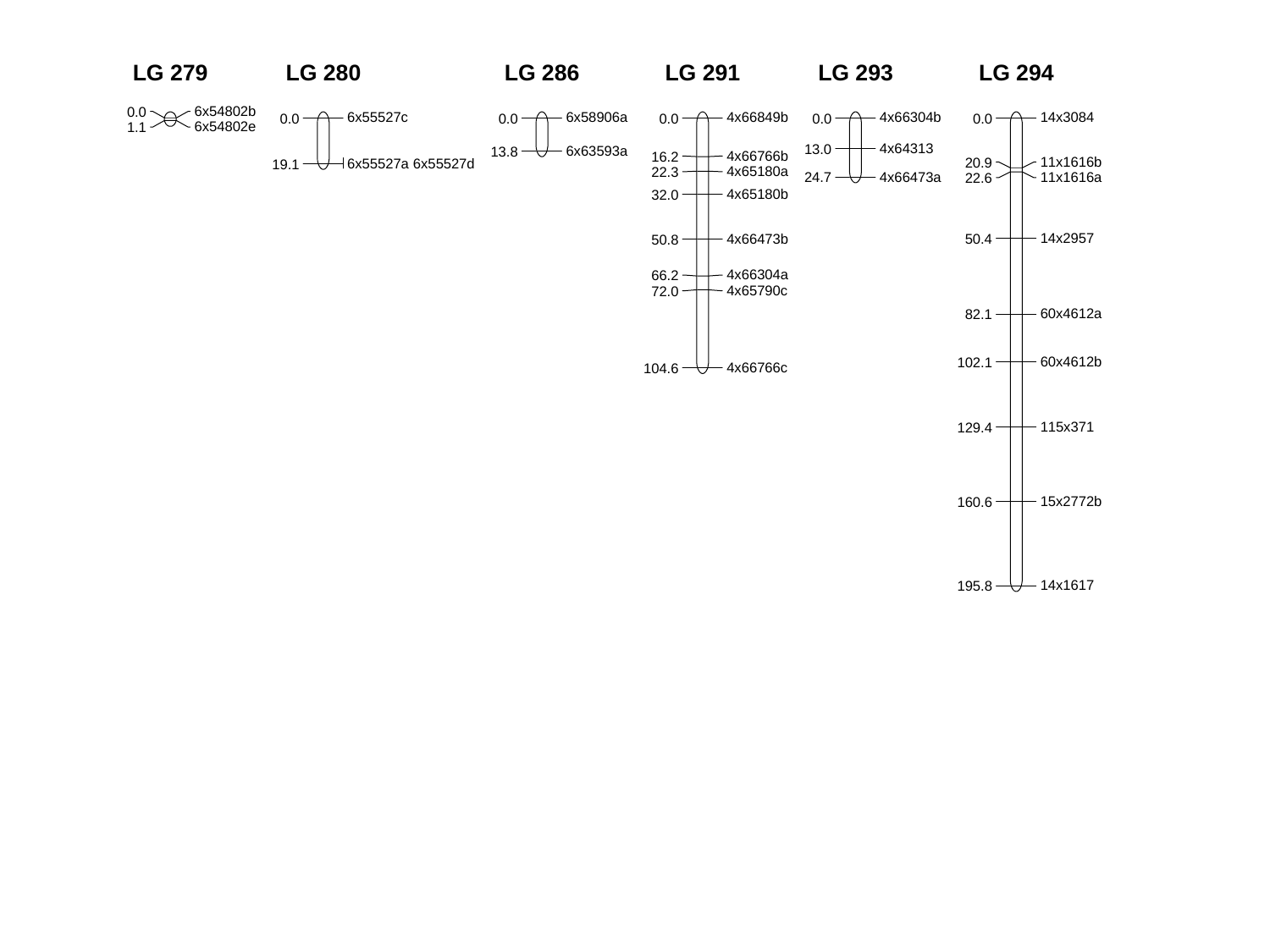

## Slide 8
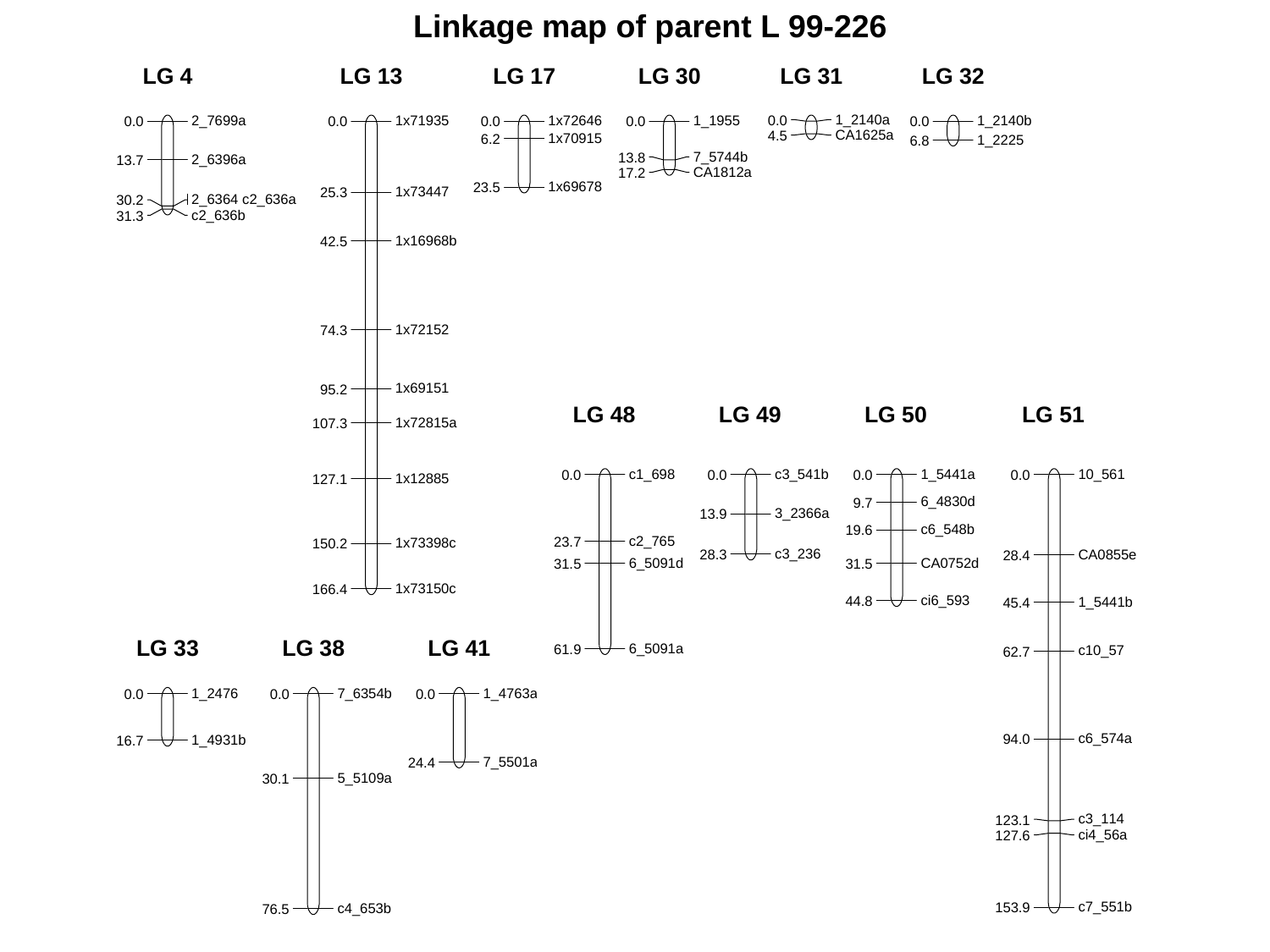

Linkage map of parent L 99-226

## Slide 9
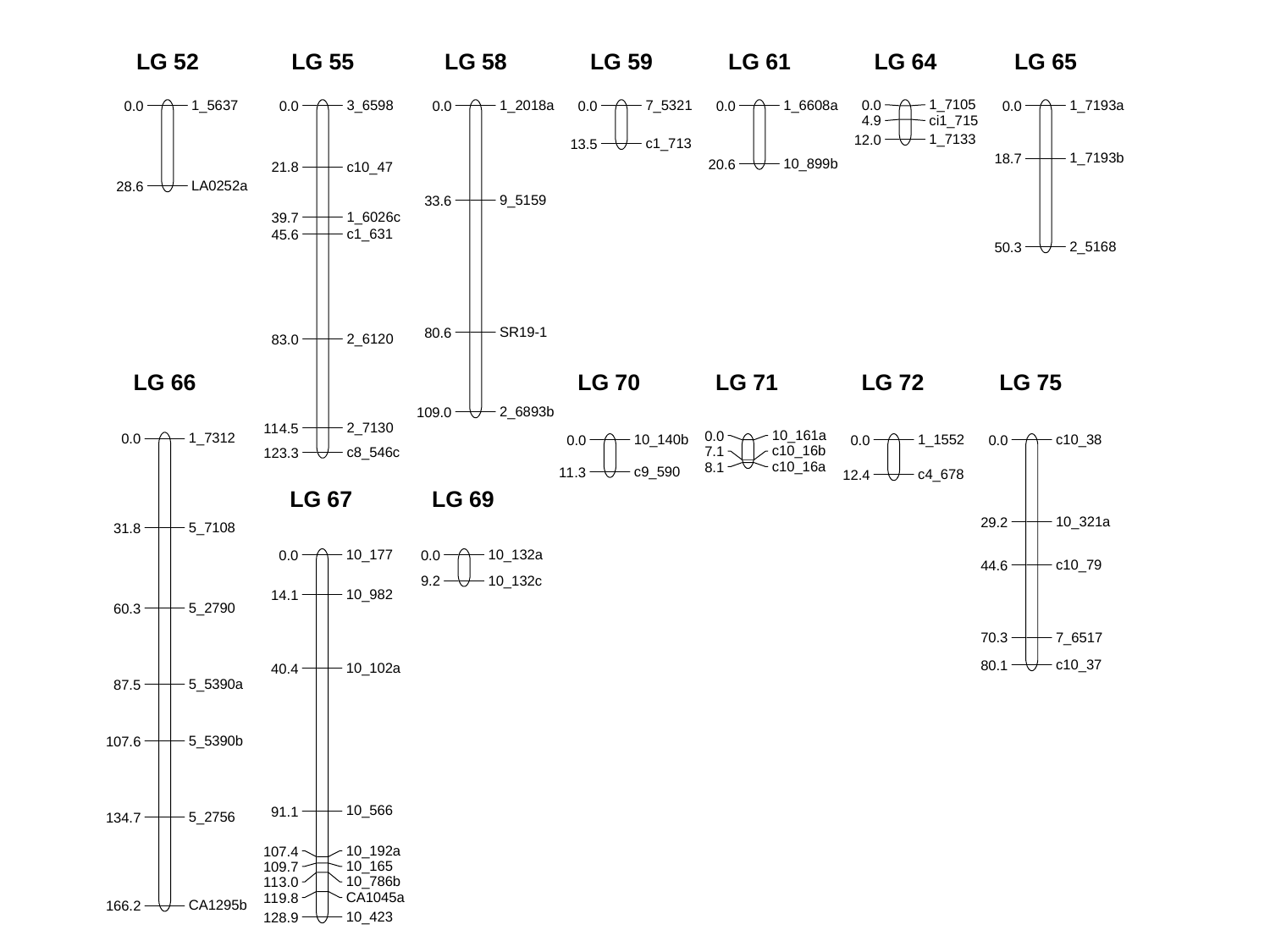

## Slide 10
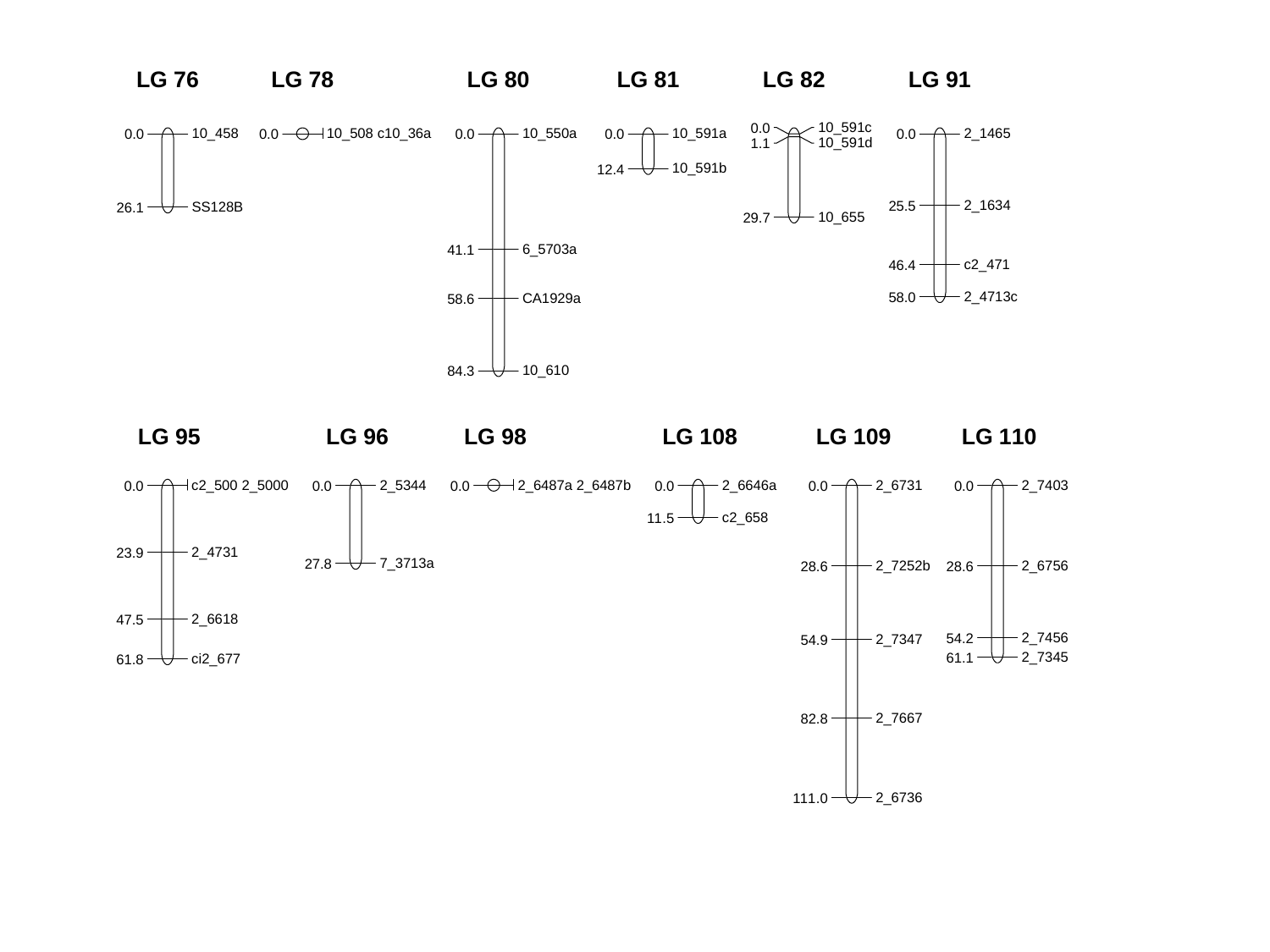

## Slide 11
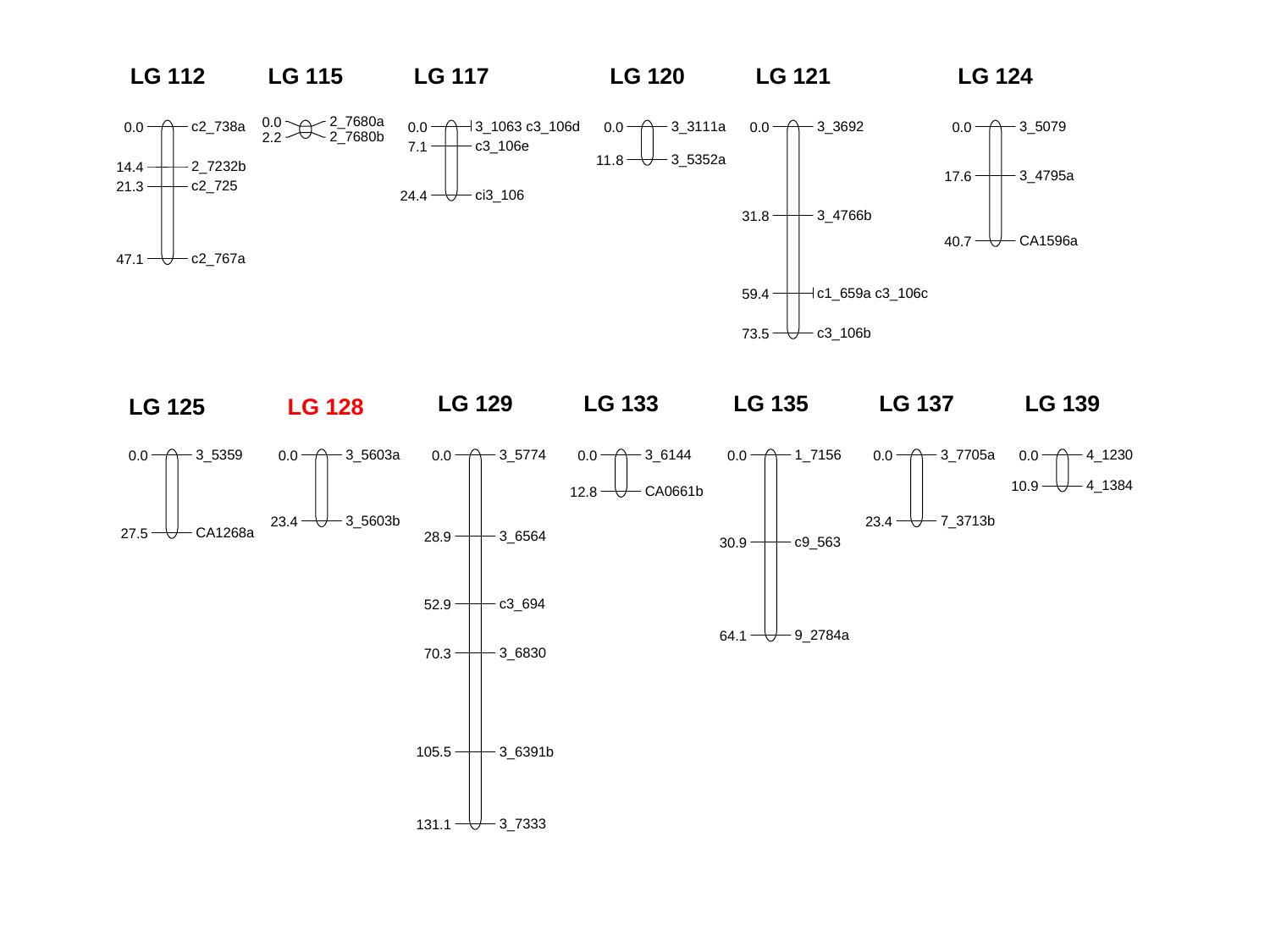

LG 125
LG 128

## Slide 12
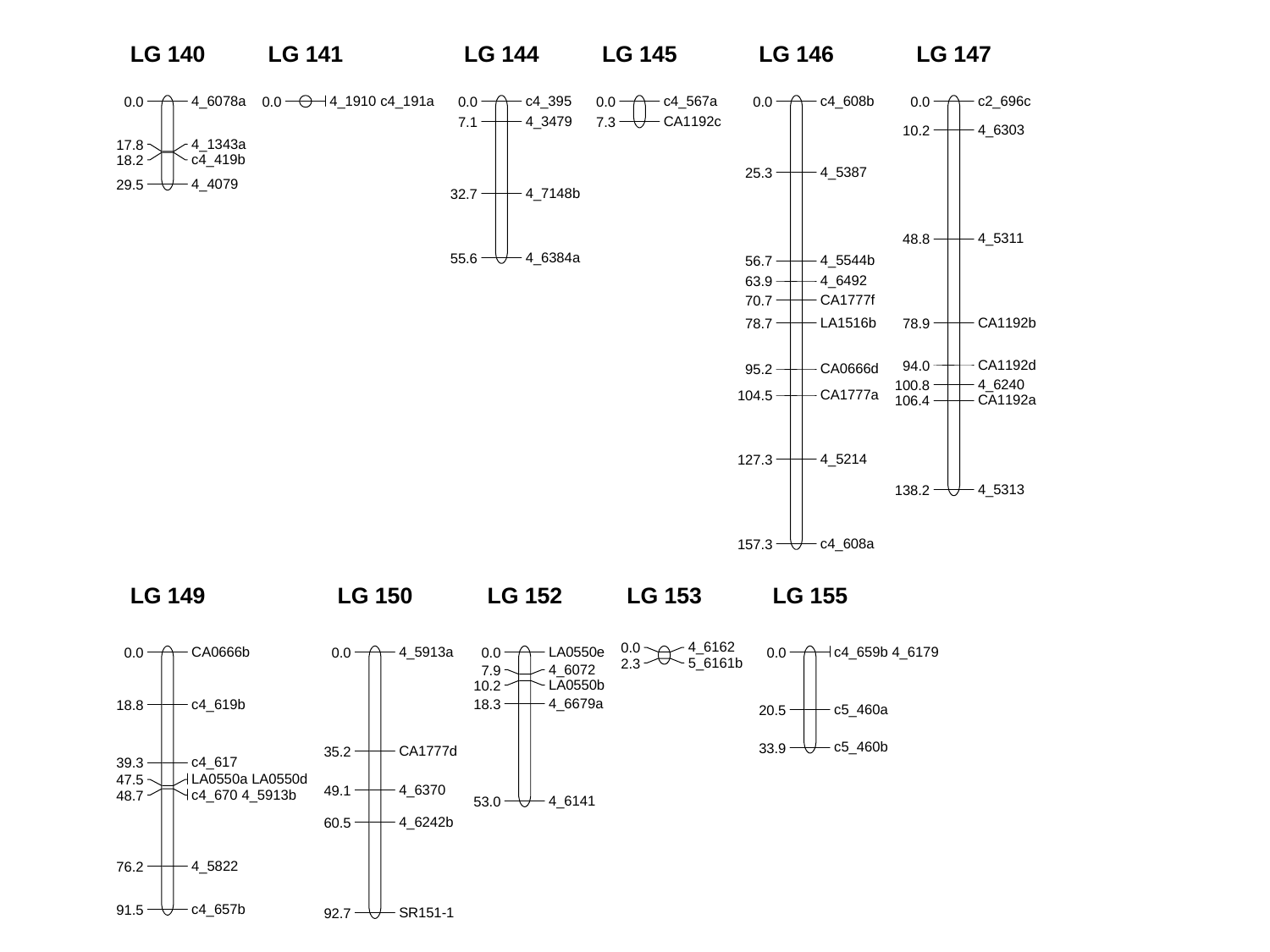

## Slide 13
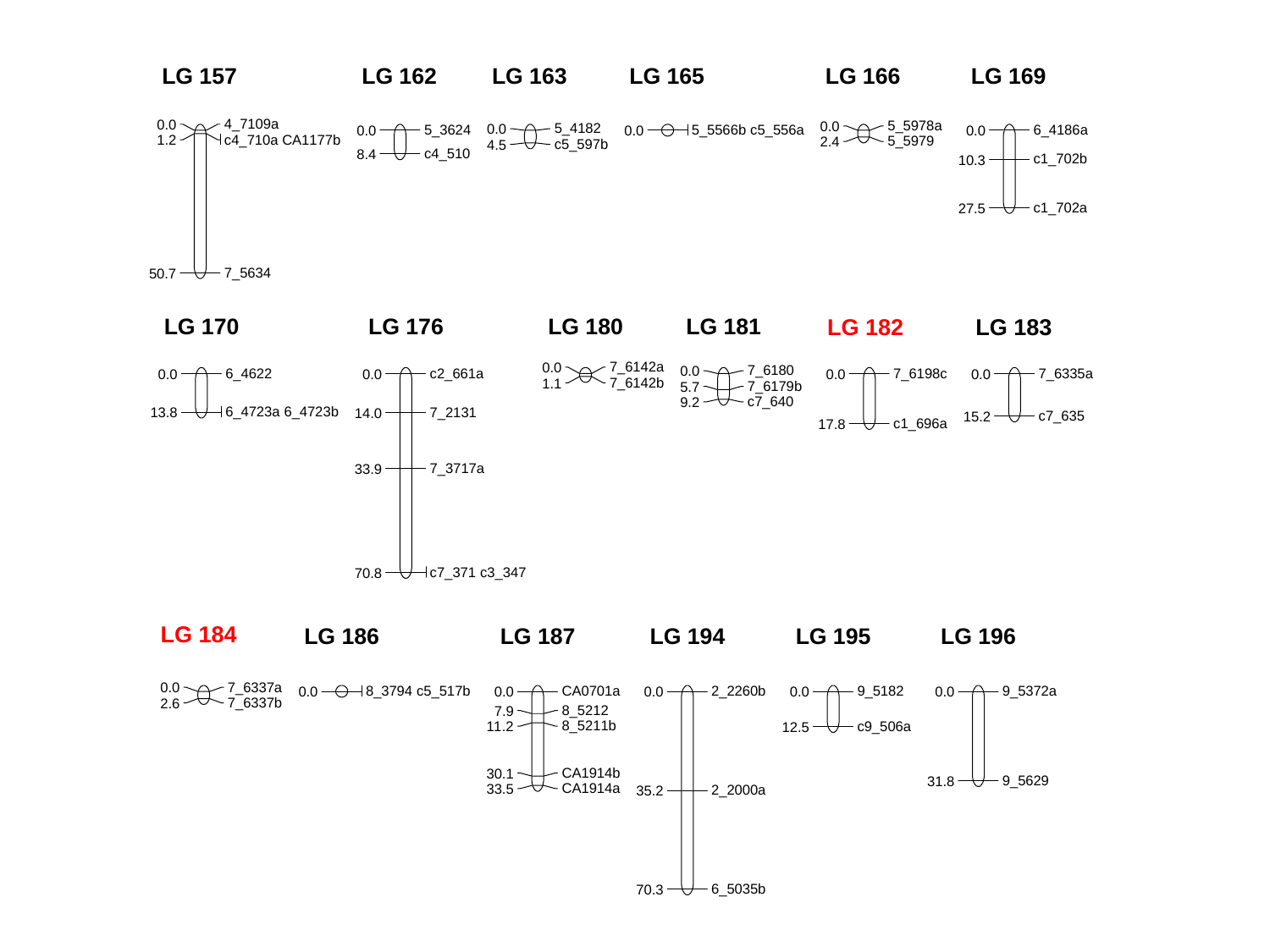

LG 182
LG 183
LG 184

## Slide 14
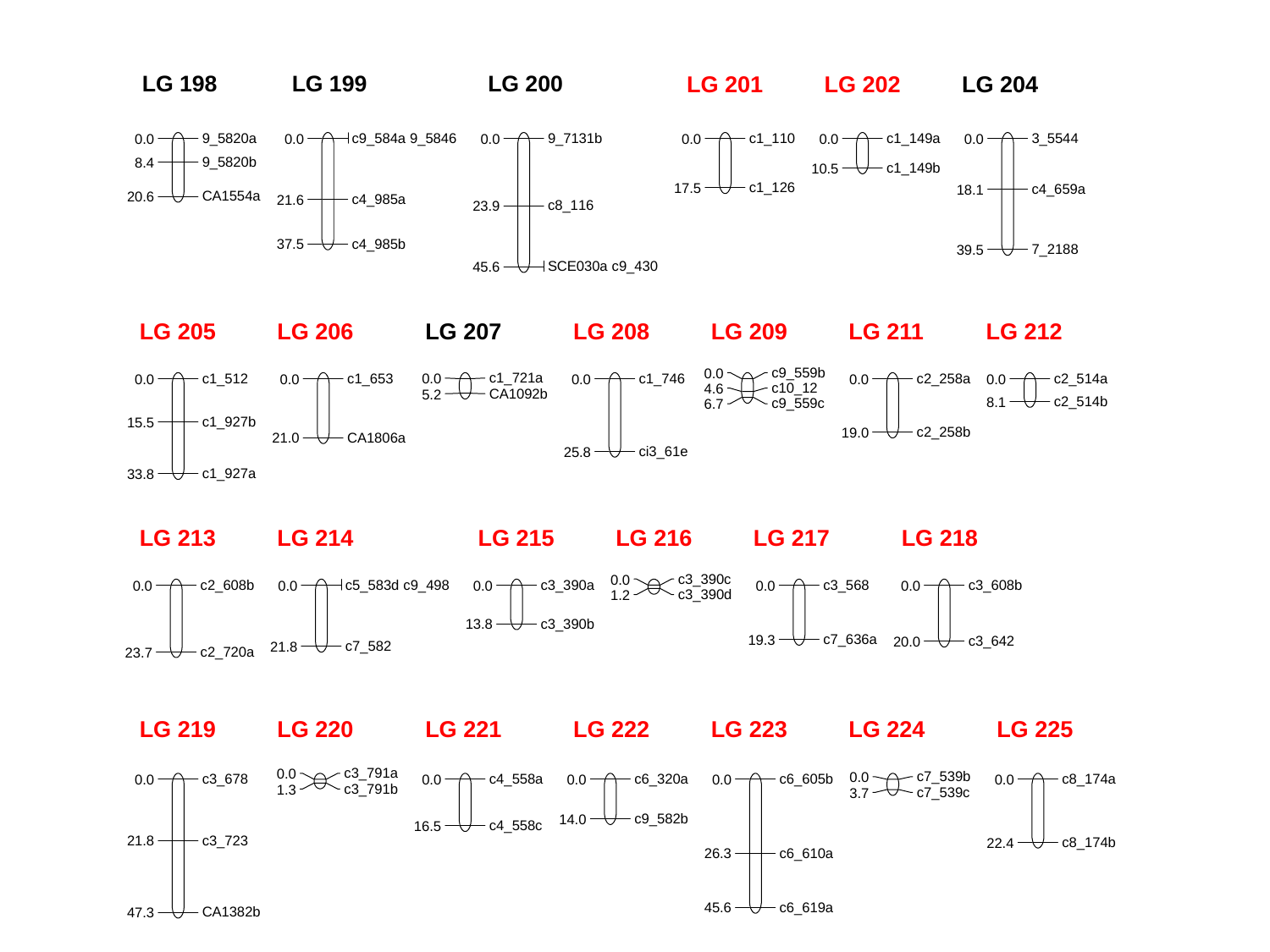

LG 201
LG 202
LG 204
LG 205
LG 206
LG 207
LG 208
LG 209
LG 211
LG 212
LG 213
LG 214
LG 215
LG 216
LG 217
LG 218
LG 219
LG 220
LG 221
LG 222
LG 223
LG 224
LG 225

## Slide 15
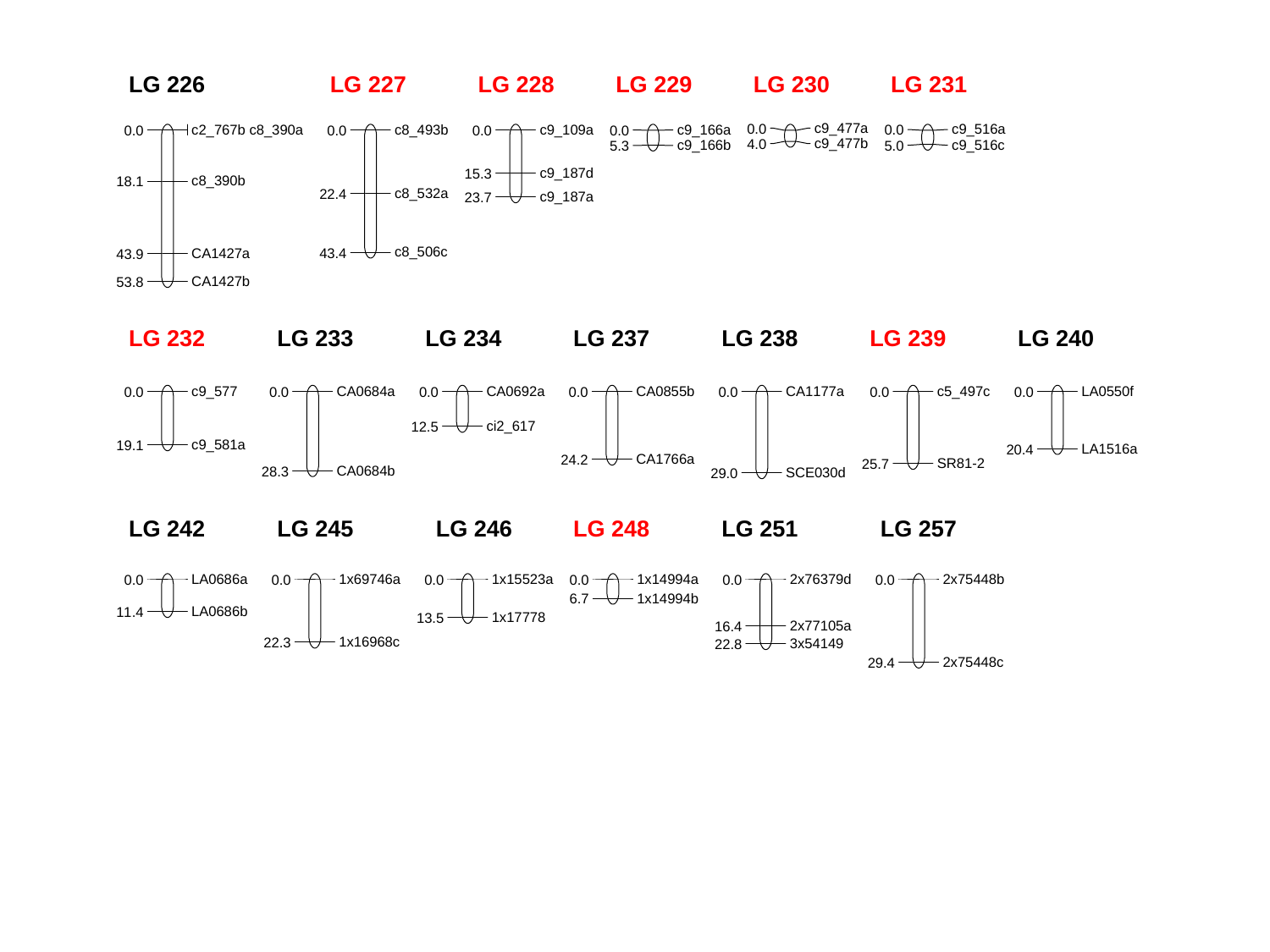

LG 226
LG 227
LG 228
LG 229
LG 230
LG 231
LG 232
LG 233
LG 234
LG 237
LG 238
LG 239
LG 240
LG 242
LG 245
LG 246
LG 248
LG 251
LG 257

## Slide 16
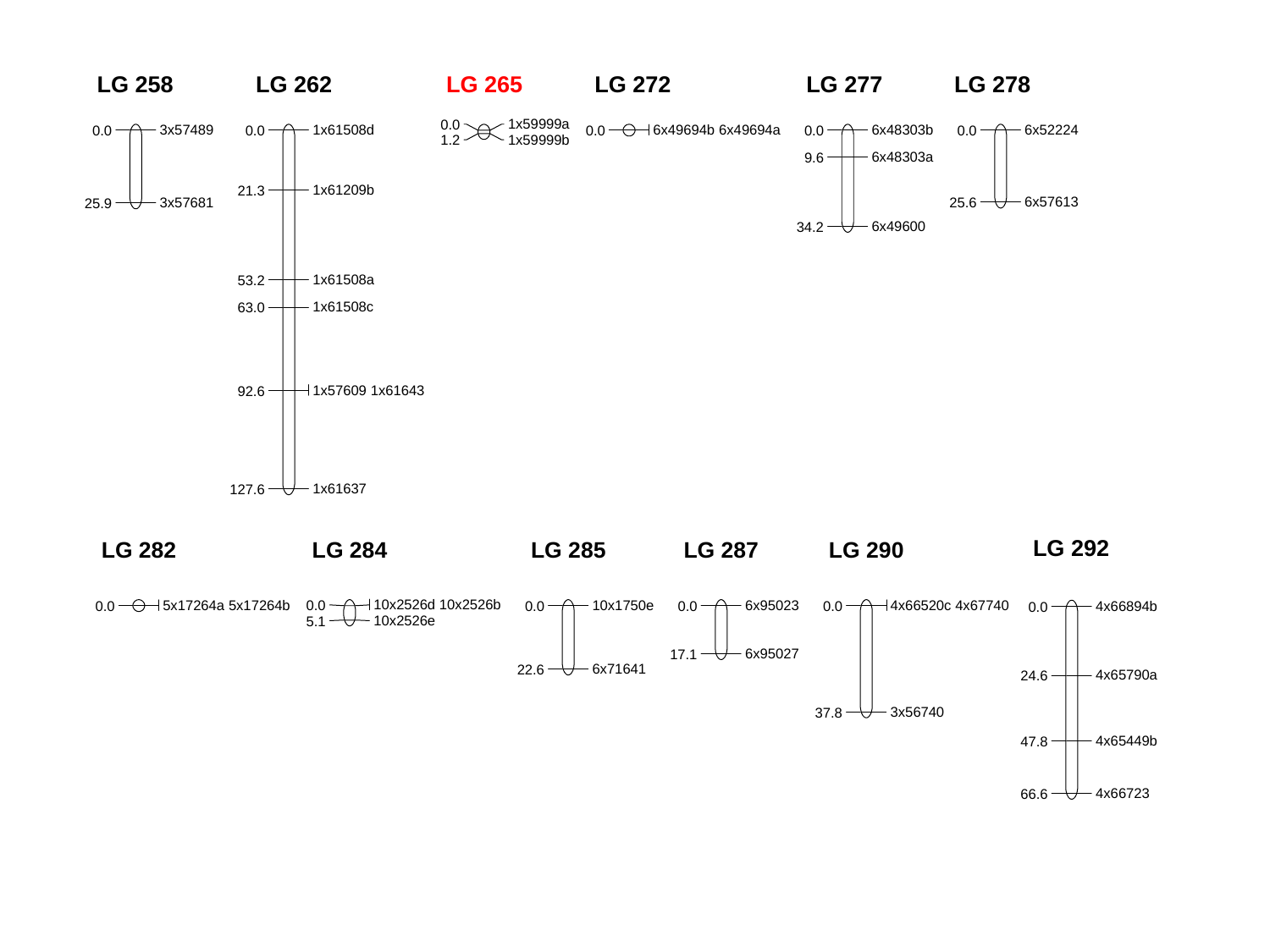

LG 258
LG 262
LG 265
LG 272
LG 277
LG 278
LG 292

## Slide 17
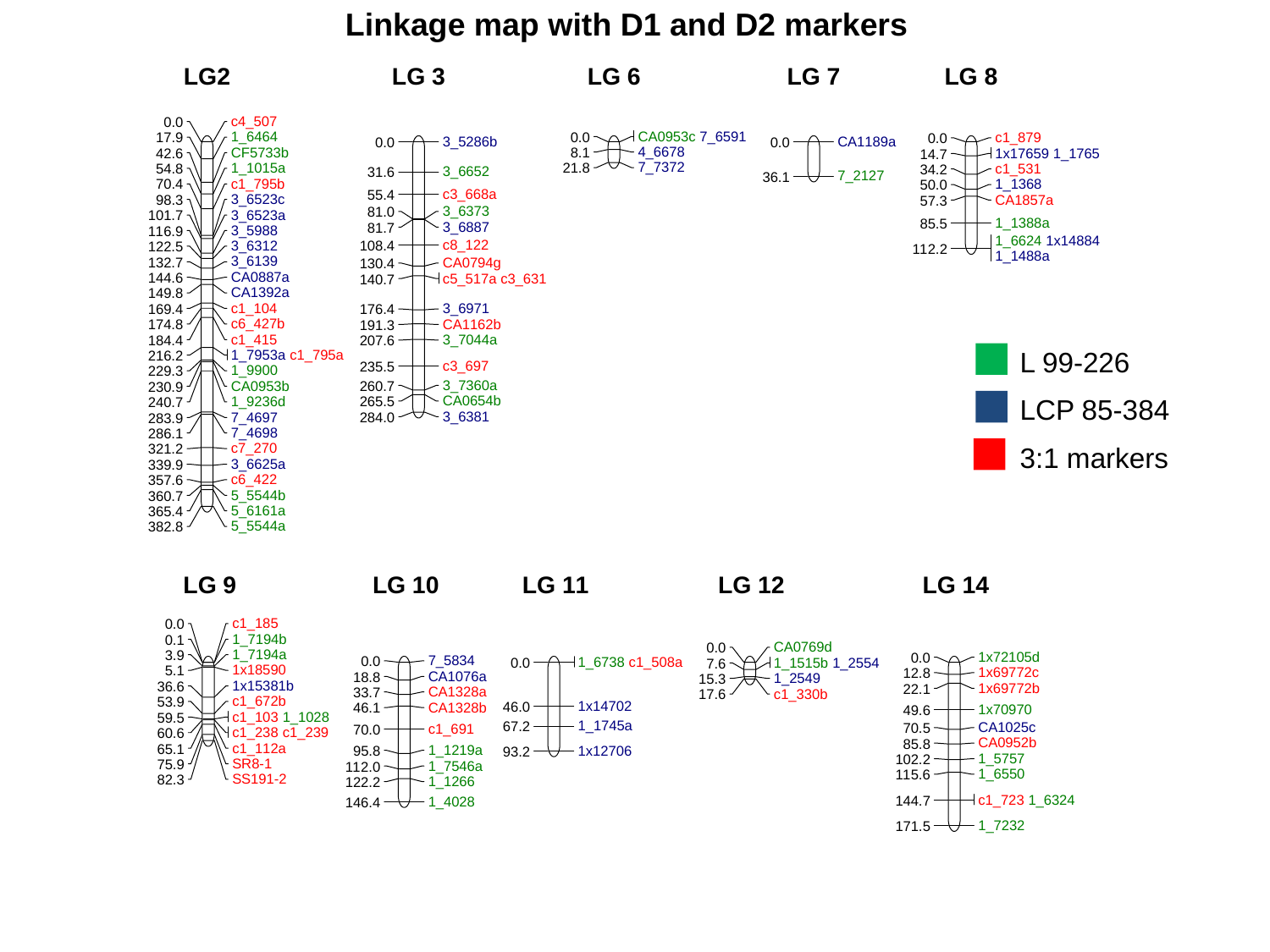

Linkage map with D1 and D2 markers
L 99-226
LCP 85-384
3:1 markers

## Slide 18
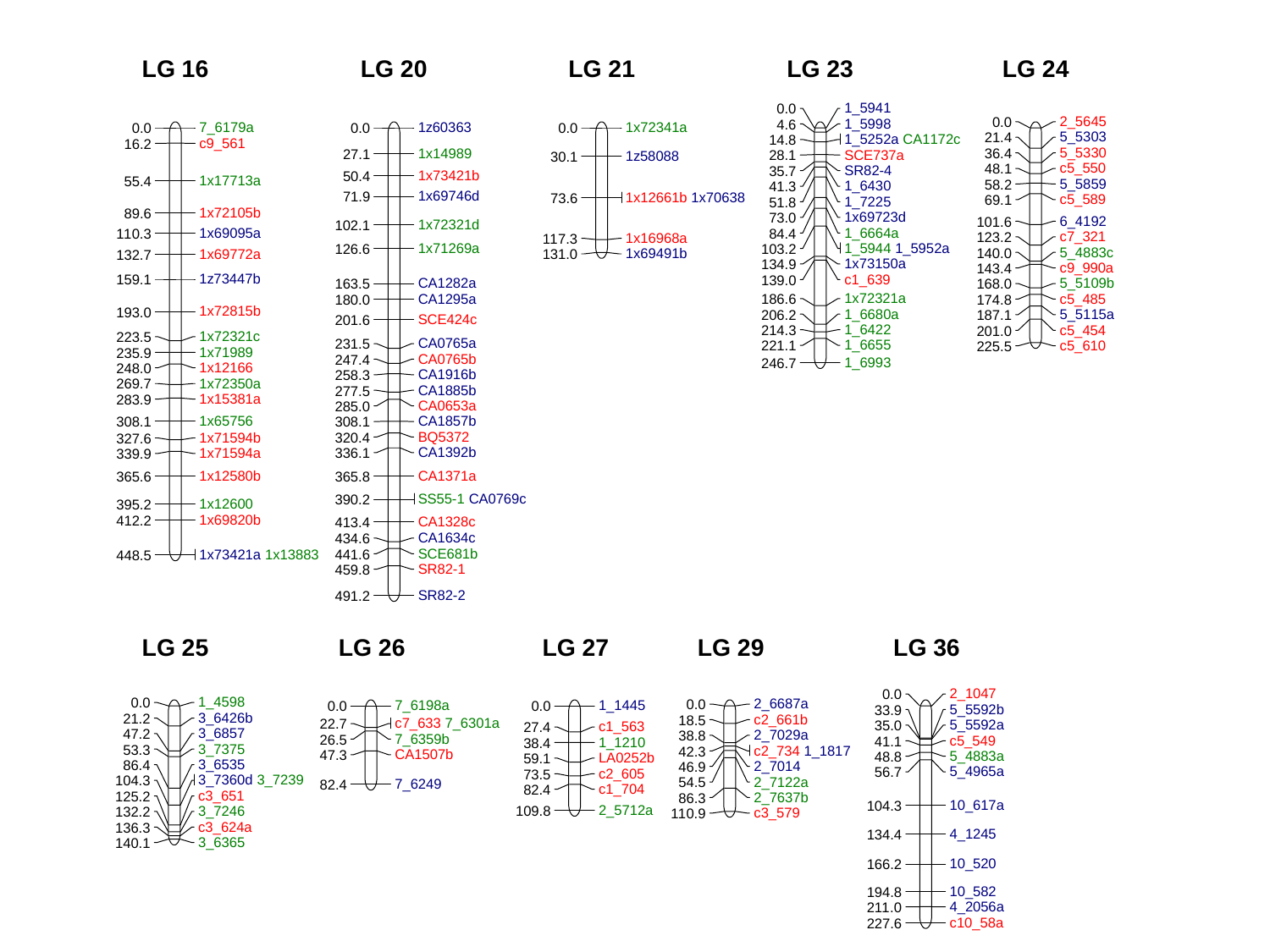

## Slide 19
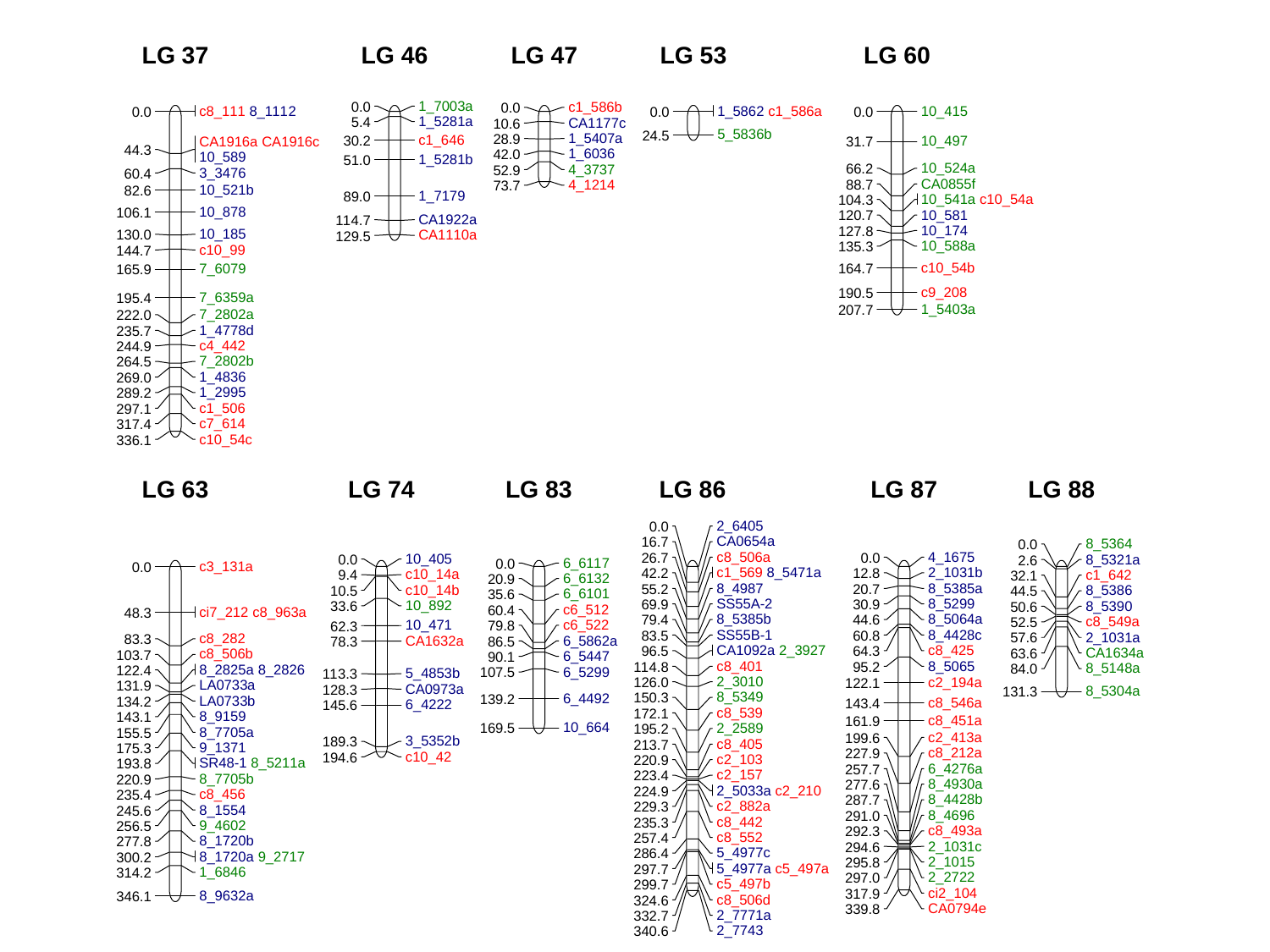

## Slide 20
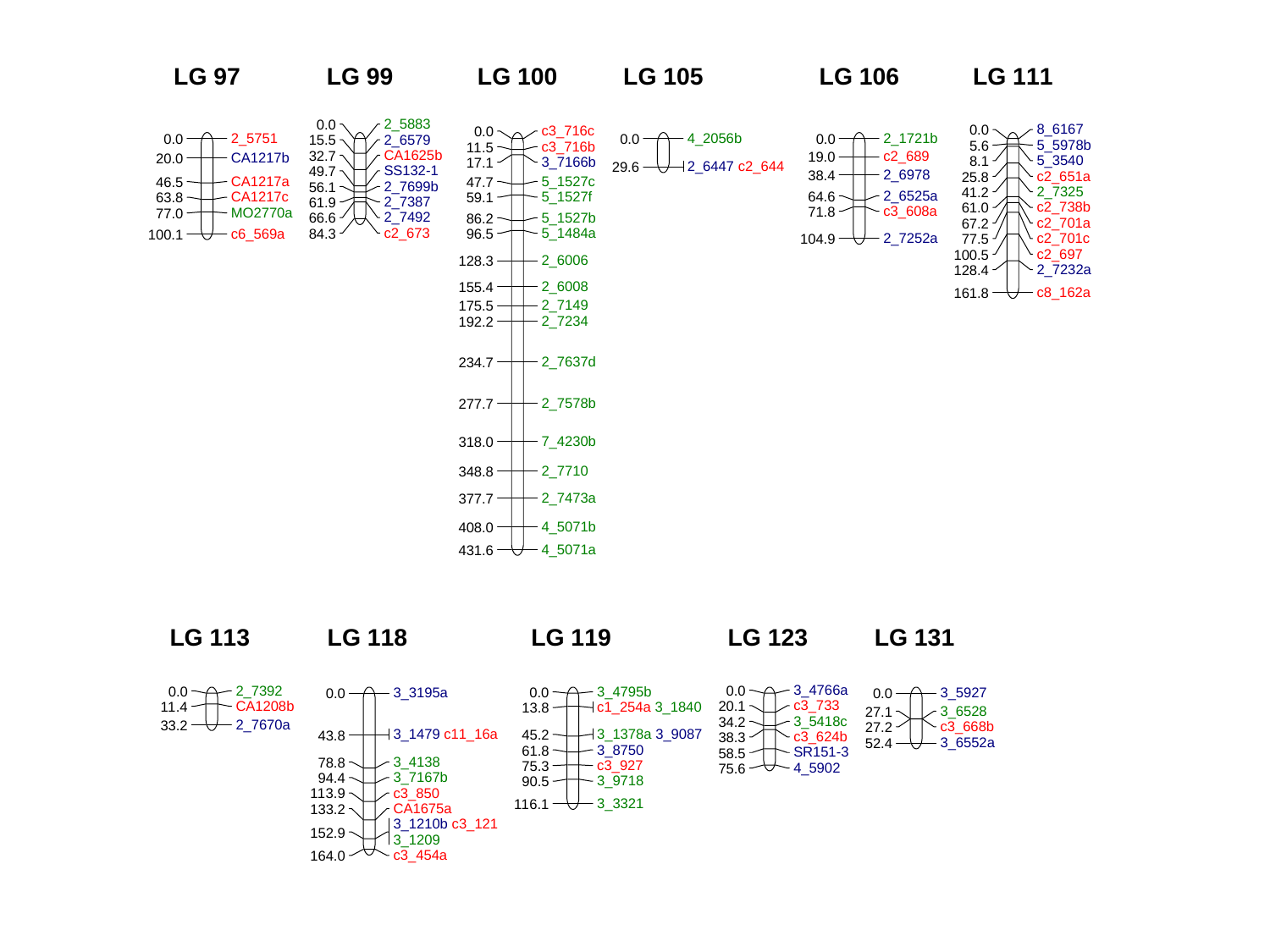

## Slide 21
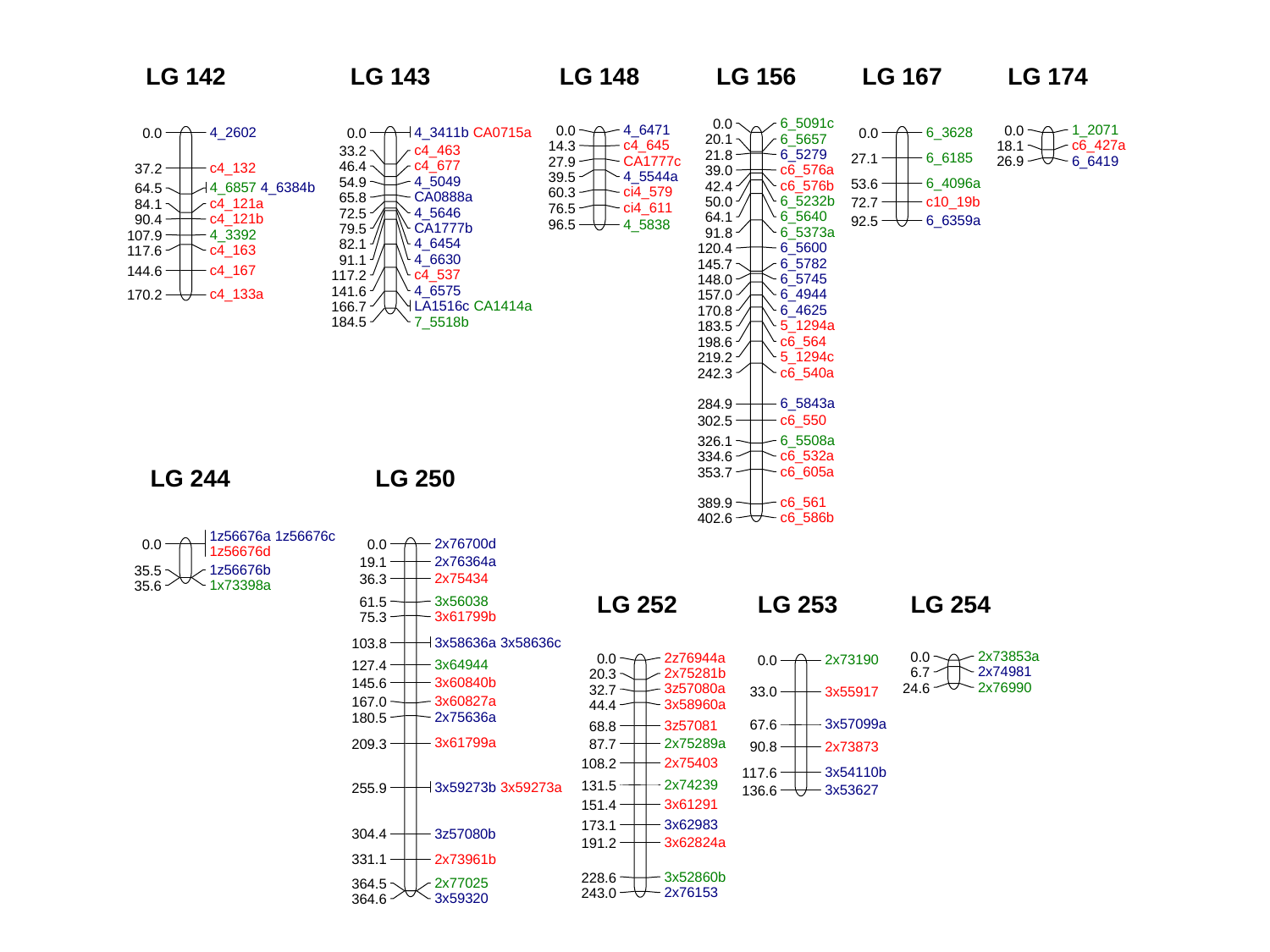

## Slide 22
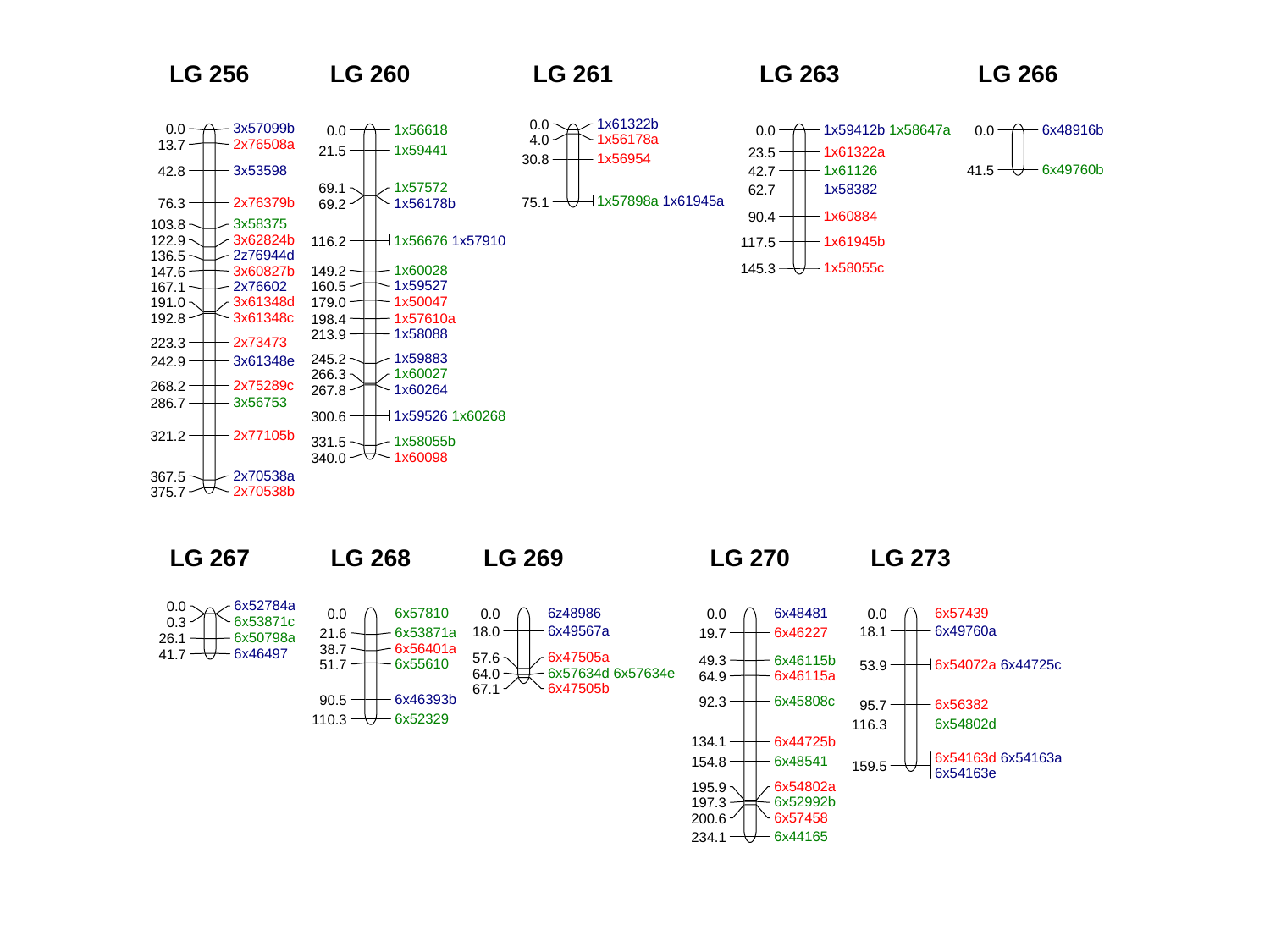

## Slide 23
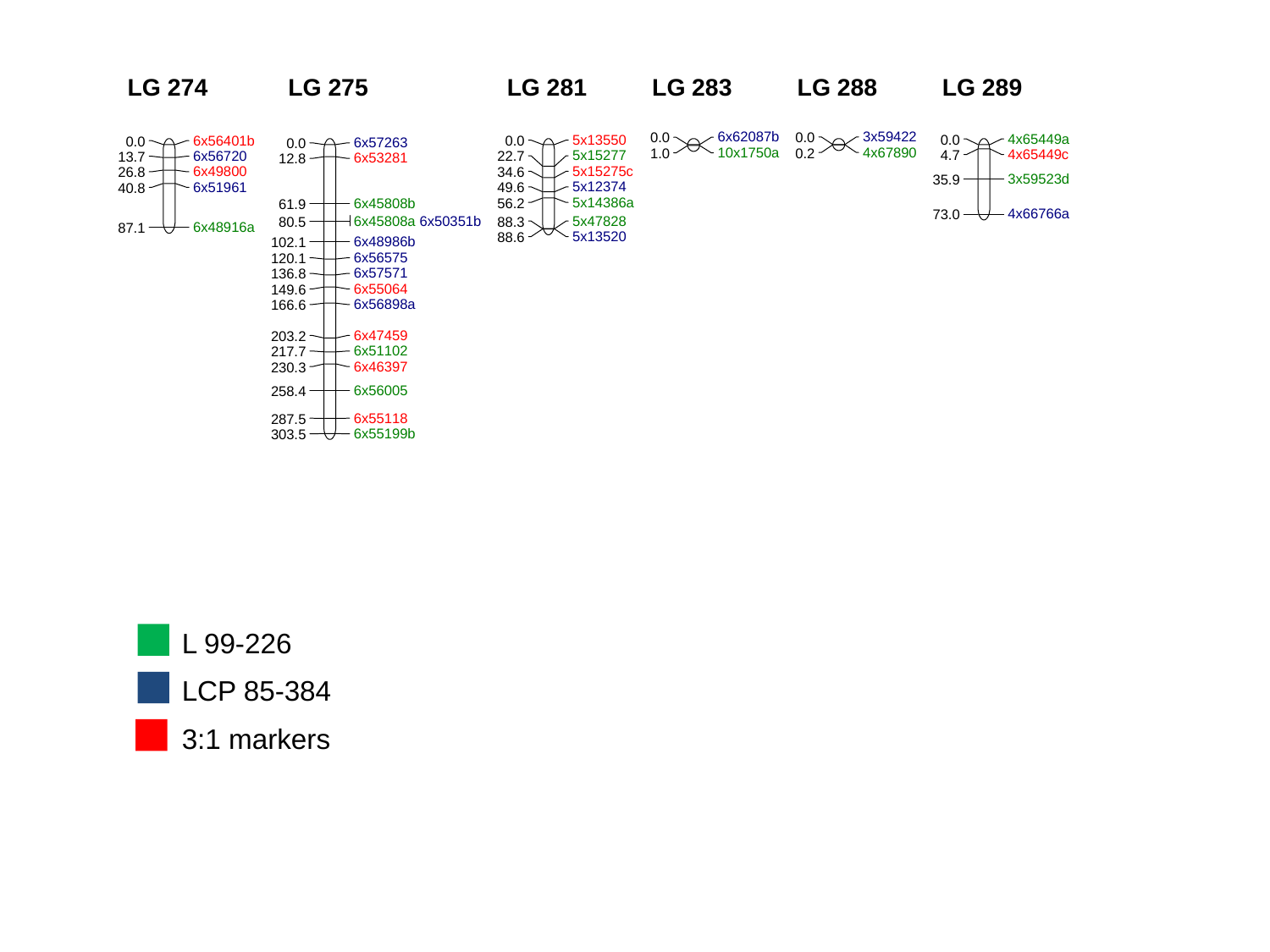

L 99-226
LCP 85-384
3:1 markers
