# Supplementary material for: Identification of Genomic Regions Controlling Leaf Scald Resistance in Sugarcane Using a Bi-parental Mapping Population and Selective Genotyping by Sequencing
Source: Front Plant Sci. 2018 Jun 26;9:877. doi: 10.3389/fpls.2018.00877 (PMC6028728; doi:10.3389/fpls.2018.00877)
Supplement: FIGURE S2 — Quantitative real-time PCR showing temporal changes in the expression of one selected gene from three QTLs in the leaf/meristematic tissues of a leaf scald resistant clone (LCP 85-384) and susceptible clone (HoCP 85-845). RNA was isolated from meristematic tissues of three independent plants (biological replicates) at 0 h (control), 24 h, 48 h, 72 h and 1 week after leaf scald inoculation. First strand synthesis, real-time PCR, and fold-change relative expression was performed as described earlier (Baisakh et al., 2012). [file Presentation_2.PPTX]

## Slide 1
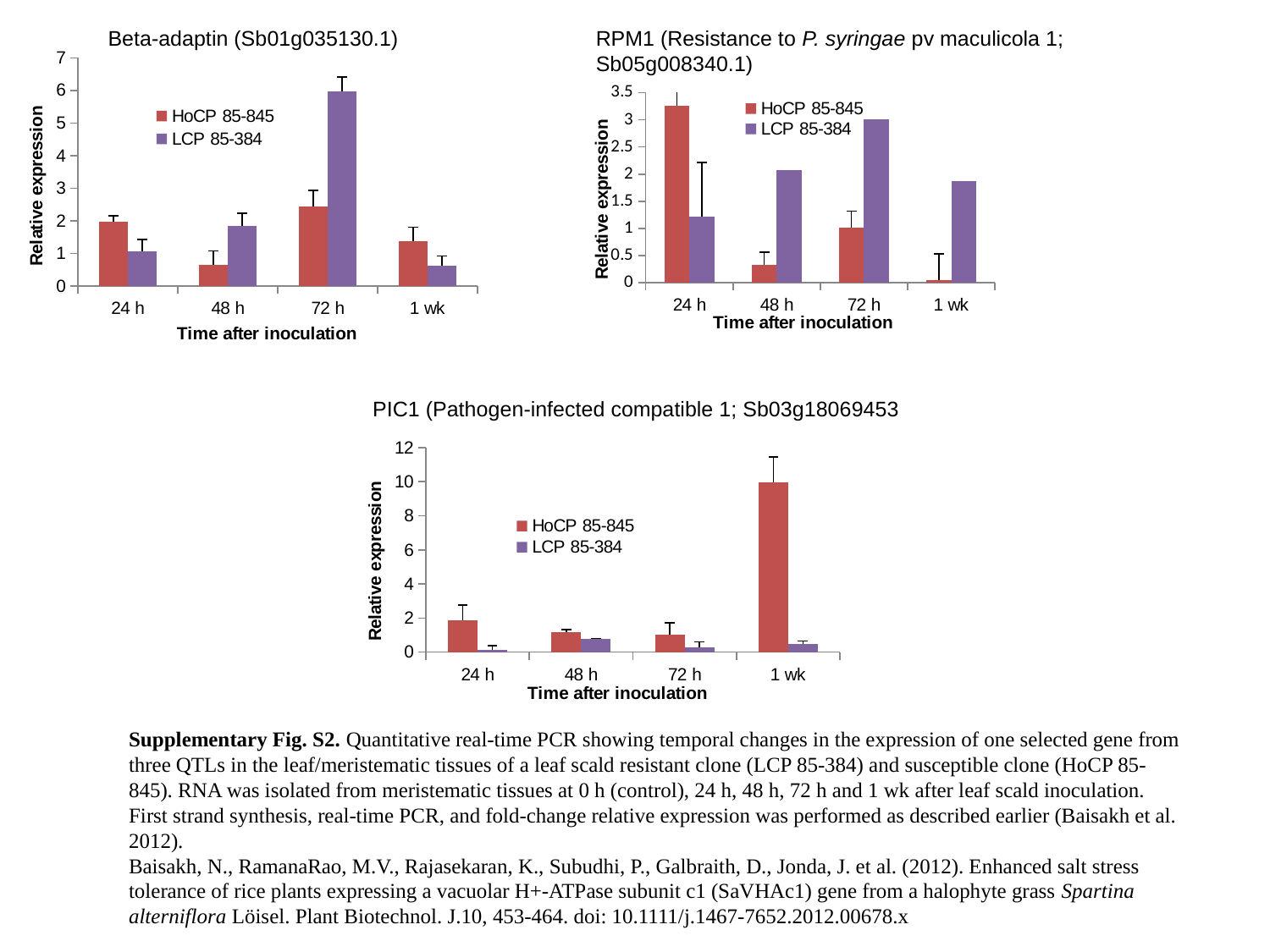

Beta-adaptin (Sb01g035130.1)
RPM1 (Resistance to P. syringae pv maculicola 1;
Sb05g008340.1)
### Chart
| Category | HoCP 85-845 | LCP 85-384 |
|---|---|---|
| 24 h | 1.971996876575349 | 1.0676262454860896 |
| 48 h | 0.6511696309975262 | 1.8370811604936719 |
| 72 h | 2.447362918225389 | 5.978310158377497 |
| 1 wk | 1.3864423867912463 | 0.6304663073948045 |
### Chart
| Category | HoCP 85-845 | LCP 85-384 |
|---|---|---|
| 24 h | 3.2620159995470126 | 1.2113335324654315 |
| 48 h | 0.3306337316770636 | 2.073967894293165 |
| 72 h | 1.0131795832495167 | 3.0037989828150624 |
| 1 wk | 0.0561310366176715 | 1.8719305420867531 |PIC1 (Pathogen-infected compatible 1; Sb03g18069453
### Chart
| Category | HoCP 85-845 | LCP 85-384 |
|---|---|---|
| 24 h | 1.8626188391538467 | 0.1538880556962506 |
| 48 h | 1.1679251536642756 | 0.7799211326433496 |
| 72 h | 1.052379516419123 | 0.2819892149961806 |
| 1 wk | 9.937856258931278 | 0.4630454020731311 |Supplementary Fig. S2. Quantitative real-time PCR showing temporal changes in the expression of one selected gene from three QTLs in the leaf/meristematic tissues of a leaf scald resistant clone (LCP 85-384) and susceptible clone (HoCP 85-845). RNA was isolated from meristematic tissues at 0 h (control), 24 h, 48 h, 72 h and 1 wk after leaf scald inoculation. First strand synthesis, real-time PCR, and fold-change relative expression was performed as described earlier (Baisakh et al. 2012).
Baisakh, N., RamanaRao, M.V., Rajasekaran, K., Subudhi, P., Galbraith, D., Jonda, J. et al. (2012). Enhanced salt stress tolerance of rice plants expressing a vacuolar H+-ATPase subunit c1 (SaVHAc1) gene from a halophyte grass Spartina alterniflora Löisel. Plant Biotechnol. J.10, 453-464. doi: 10.1111/j.1467-7652.2012.00678.x
