# Supplementary material for: Identification of Genomic Regions Controlling Leaf Scald Resistance in Sugarcane Using a Bi-parental Mapping Population and Selective Genotyping by Sequencing
Source: Front Plant Sci. 2018 Jun 26;9:877. doi: 10.3389/fpls.2018.00877 (PMC6028728; doi:10.3389/fpls.2018.00877)
Supplement: TABLE S1 — Single dose markers associated with the leaf scald response in the LCP85-384 × L 99-226 F1 population as identified by single marker analysis. [file Table_1.DOCX]

**Supplementary Table S1**. Single dose markers associated with the leaf scald response in the LCP85-384 x L 99-226 F1 population as identified by single marker analysis

| Marker | LG | Position | LOD | PVE (%) | Add | Dom |
| --- | --- | --- | --- | --- | --- | --- |
| c3_579 | 29 | 110.8811 | 2.3 | 9.42 | -0.19 | -0.11 |
| c1_586b | 47 | 0 | 2.6 | 12.34 | -0.19 | -0.25 |
| c3_689b | 104 | 18.4006 | 3.7 | 17.27 | -0.18 | 0.32 |
| 6x57571 | 275 | 136.8225 | 2.6 | 12.44 | 0.25 | -0.07 |
