# Supplementary material for: Identification of Genomic Regions Controlling Leaf Scald Resistance in Sugarcane Using a Bi-parental Mapping Population and Selective Genotyping by Sequencing
Source: Front Plant Sci. 2018 Jun 26;9:877. doi: 10.3389/fpls.2018.00877 (PMC6028728; doi:10.3389/fpls.2018.00877)
Supplement: TABLE S2 — Regression analysis showing contribution of individual SNP markers associated with leaf scald resistance on 186 progeny of the LCP85-384 × L 99-226 F1 population. [file Table_2.DOCX]

**Supplementary Table S2**. Regression analysis showing contribution of individual SNP markers associated with leaf scald resistance on 186 progeny of the LCP85-384 x L 99-226 F1 population

| Marker | LG | b0 | b1 | -2ln(L0/L1) | F(1,n-2) | pr(F) | R^2^ |
| --- | --- | --- | --- | --- | --- | --- | --- |
| 5-1527e | 77 | 0.583 | 0.313 | 15.355 | 15.888 | 0.0001*** | 0.09 |
| c3-689b | 104 | 0.426 | 0.465 | 6.921 | 6.982 | 0.0090** | 0.04 |
| c3-579 | 29 | 1.918 | 0.526 | 4.916 | 4.929 | 0.0278* | 0.03 |
