# Supplementary material for: Identification of Genomic Regions Controlling Leaf Scald Resistance in Sugarcane Using a Bi-parental Mapping Population and Selective Genotyping by Sequencing
Source: Front Plant Sci. 2018 Jun 26;9:877. doi: 10.3389/fpls.2018.00877 (PMC6028728; doi:10.3389/fpls.2018.00877)
Supplement: TABLE S3 — Genes known to be involved in biotic stress response, which are close to the QTLs identified, and used for expression analysis. [file Table_3.DOCX]

**Supplementary Table S3**. Genes known to be involved in biotic stress response, which are close to the QTLs identified, and used for expression analysis

| Marker | LG | Chr | position | Locus ID | EST ID | Gene annotation |
| --- | --- | --- | --- | --- | --- | --- |
| 5_1527g | 77 | 5 | 15275658 | Sb05g008340.1 | Sb05g008340.1 | nucleotide binding/protein binding  (RPM1;  Resistance to *P. syringae* pv maculicola 1) |
| 3_579 | 29 | 3 | 57957141 | Sb03g18069453 | SCJFHR1033E05.b | Pathogen-infected compatible 1 (PIC1) |
| 1_586 | 47 | 1 | 58647793 | Sb01g035130.1 | SCAGFL8011G05.g | Beta-adaptin, putative |
